# Molecular characterization of four *Helicobacter cetorum* strains from dolphins compared to human *Helicobacter pylori*

**DOI:** 10.1080/19490976.2025.2557982

**Published:** 2025-09-25

**Authors:** Bodo Linz, Nicole Tegtmeyer, Sharmin Afroz, Mathias Müsken, James G. Fox, Freddy Haesebrouck, Mou-Chieh Kao, Heinrich Sticht, Steffen Backert

**Affiliations:** a Division of Microbiology, Department Biology, Friedrich-Alexander Universität Erlangen- Nürnberg, Erlangen, Germany; b Central Facility for Microscopy, Helmholtz Centre for Infection Research, Braunschweig, Germany; c Department of Biological Engineering, Massachusetts Institute of Technology, Cambridge, MA, USA; d Department of Pathobiology, Pharmacology and Zoological Medicine, Faculty of Veterinary Medicine, Ghent University, Merelbeke, Belgium; e Institute of Molecular Medicine, National Tsing Hua University, Hsinchu, Taiwan; f Division of Bioinformatics, Institute of Biochemistry, Friedrich-Alexander-Universität Erlangen-Nürnberg, Erlangen, Germany

**Keywords:** *Helicobacter cetorum*, *Helicobacter pylori*, VacA, urease, serine protease HtrA, evolution

## Abstract

*Helicobacter* species colonize the stomachs of many aquatic and terrestrial mammals, including *Helicobacter pylori* in humans and *Helicobacter cetorum* in dolphins. There are several *H. cetorum* genome sequences in databases, but a detailed molecular characterization of these bacteria is missing. Here, we compared four *H. cetorum* isolates from dolphins with *H. pylori* strains using electron microscopy as well as structural and functional analyses. All strains expressed similarly high urease activity and were hemolytic to erythrocytes. Western blots revealed conserved expression of flagellin-A, neutrophil-activating protein NapA, serine protease HtrA, γ-glutamyl-transpeptidase GGT and toxin VacA. In contrast, the virulence-associated *cag* pathogenicity island of *H. pylori* is missing in *H. cetorum*. 3D-modeling revealed similar structures of hexameric VacA from both species with minor differences. *H. cetorum* VacA expression was associated with vacuole formation in epithelial cells similar to that of *s1/m2*, but not as strong as *H. pylori s1/m1 vacA* strains, and complementation of *H. pylori* with *H. cetorum vacA* restored the *s1/m2*-like VacA phenotype. While *H. pylori* infection robustly activated toll-like receptors TLR1, TLR2, TLR4, TLR5, TLR9, and TLR10, *H. cetorum* only stimulated TLR1/2, TLR4, and TLR10, but much less pronounced than *H. pylori*. Accordingly, infection of epithelial cells with *H. pylori* induced strong DNA damage, NF-κB activation, and IL-8 secretion, but these responses were barely detectable in *H. cetorum*-infected cells. Activation of only few TLRs and significantly weaker pro-inflammatory responses than *H. pylori* suggest that *H. cetorum* is a commensal or only moderately virulent pathobiont in the stomach of dolphins, comparable to the less pathogenic *cag*PAI-negative *H. pylori* strains in humans. Since *H. cetorum* is evolutionarily older than *H. pylori*, we propose that *H. cetorum* represents a direct ancestor of *H. pylori* that arose after a host jump over 623,000 years ago, which is the coalescence time of the two species.

## Introduction

Many *Helicobacter* species share a long co-evolution with their mammalian hosts. The well-characterized human stomach bacterium *Helicobacter pylori* has been associated with modern humans since their origin in Africa about 200,000 years ago.^1,2^ This infection is usually acquired in childhood when the immune system is not yet fully developed and the acidity of the stomach is less pronounced than in adulthood. While transmission of the bacteria commonly occurs within families, extra-familial transmission between unrelated individuals has also been reported, particularly in the developing world.^3,4^ During the long co-evolution, host and pathogen diversified, which resulted in the development of distinct biogeographic *H. pylori* populations in Africa (hpAfrica1, hpAfrica2, hpNEAfrica), Eurasia (hpEastAsia, hpAsia2, hpEurope), the Sahul (hpSahul), Siberia and Americas (hpNorthAsia).^5–8^

Infection with *H. pylori* is widespread; about 50% of the human global population is estimated to be colonized by this bacterium.^9^ Colonization of the highly acidic stomach is facilitated by the release of urease and by flagella. Secreted urease buffers the immediate surrounding of the bacteria by hydrolysis of urea into ammonium and carbon dioxide.^10^ Flagella, that are encoded by a large set of flagellar genes, including the flagellin gene *flaA*, enable the bacteria to migrate through the mucus layer of the epithelium.^11^ Adherence to the gastric mucosa is then mediated by bacterial adhesins such as the blood group antigen-binding proteins BabA and BabB,^12^ the sialic acid-binding adherence protein SabA,^13^ and the HopQ adhesin that binds the human carcinoembryonic antigen-related cell adhesion molecule (CEACAM) receptors.^14,15^ During the initial colonization, *H. pylori* causes acute gastric inflammation in all infected individuals.^16^ The inflammation then generally subsides in the majority of the infected people and the subsequent chronic infection remains asymptomatic. However, persistent infection and inflammation can lead to the development of gastric and duodenal ulcers in approximately 10–15% of the infected individuals and gastric adenocarcinoma or lymphoma of the mucosa-associated tissue (MALT) in about 1%, usually late in life.^17^ Conversely, *H. pylori* colonization was reported to be beneficial early in life by protecting the human host from autoimmune diseases such as asthma and inflammatory bowel disease.^18^

The development of malignant disease depends on several factors, including the genetic susceptibility, lifestyle and diet of the host, and the genotype of the pathogen, particularly of its virulence genes.^19^ Important *H. pylori* virulence factors include the *cag* pathogenicity island (*cag*PAI) and its effector protein CagA that are only present in the highly virulent type-I strains, but not in the less virulent type-II strains, the vacuolating cytotoxin VacA, serine protease HtrA, and a variety of outer membrane proteins that are involved in host cell binding.^20^ The *cag*PAI encodes a type IV secretion system (T4SS) that injects the effector molecules CagA and ADP-heptose (ADPH) into gastric epithelial cells.^21,22^ Once injected, ADPH activates pro-inflammatory responses by stimulating transcription factor NF-κB and the release of interleukin-8 (IL-8),^22^ and CagA interferes with various intracellular signaling pathways, thus inducing malignant cell changes.^23^ T4SS assembly and CagA delivery occur at the basolateral side of the epithelial cells, which requires opening of the cell-to-cell junctions. Cleavage of junction proteins by *H. pylori* serine protease HtrA enables bacterial migration between the cells and subsequent T4SS functions.^24^ A serine/leucine polymorphism (171S/L) affects the stability of proteolytically active HtrA trimers, and thus the disruption of cell junctions and subsequent CagA delivery. Strains with the 171 L-type HtrA, which are evolutionarily ancestral, cause stronger cleavage of the junction proteins and more frequent DNA damage in host chromosomes.^25–27^ Another important virulence factor is the vacuolating cytotoxin VacA that induces the formation of large cellular vacuoles from lysosomes, alters various cellular signaling pathways, and interferes with immune cell functions.^28,29^ In addition, VacA triggers membrane disruption in mitochondria and activates apoptosis of infected cells.^30^ Interestingly, CagA and VacA display antagonistic effects on actin-cytoskeletal rearrangements and apoptosis of infected host cells.^31,32^ Although the *vacA* gene is present in all *H. pylori* isolates, strains differ in VacA expression, secretion, and cytotoxic activity. VacA cytotoxicity is determined by allelic variations in heterogenic regions of the protein, most notably the signal peptide region (alleles *s1, s2*) that determines vacuole formation and the middle region (*m1, m2*) that is thought to be associated with cell binding.^28^ Allele *s1* facilitates the vacuole-forming activity by creating an anion channel across the lysosome membrane. In contrast, *s2* VacA fails to form anion channels across the lipid bilayers because of a hydrophilic 12-amino-acid extension in the signal peptide region that prevents integration into the hydrophobic membrane. As a result, *s1/m1* VacA and to a lesser degree *s1/m2* VacA are cytotoxic to epithelial cells, but *s2/m2* VacA does not display cytotoxicity.^29,33^ The *s2/m1* allele combination is extremely rare. The individual VacA alleles are associated with the presence of the *cag*PAI. Strains that contain the *cag*PAI (type I strains) usually contain *s1/m1* or *s1/m2* VacA variants, whereas *cag*PAI-negative (type II) strains contain the non-cytotoxic *s2/m2* type VacA.^20^

**Figure 1.**
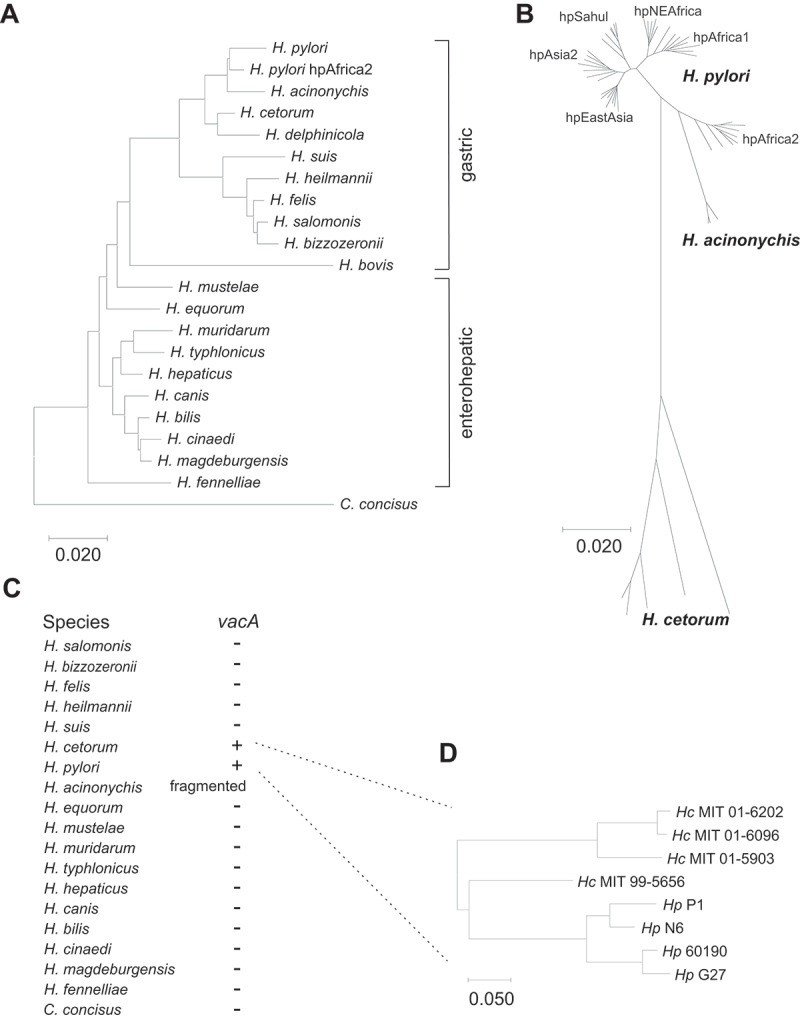
*16SrRNA* gene and housekeeping gene-based phylogeny and presence of the *vacA* gene in gastric *Helicobacter* species. A) Neighbor-joining tree of *16SrRNA* gene sequences. B) Neighbor-joining tree of concatenated housekeeping gene sequences *atpA*, *efp*, *mutY*, *ppa*, *trpC*, and *ureI* from *H. pylori* and its closest relatives *H. acinonychis* and *H. cetorum*. Species names are in black, and *H. pylori* populations are in gray. C) presence and absence of the *vacA* gene. D) phylogeny of VacA protein sequences from four *H. pylori* and four *H. cetorum* strains. *Hc* - *H. cetorum*; *Hp* - *H. pylori*.

The closest known relative of *H. pylori* is *H. acinonychis* (Figure 1), which has been isolated from large felines living in captivity, but not from wild animals. *H. acinonychis* infection and erosions of the gastric epithelium, including chronic gastritis and ulcers, have been reported in captive cheetahs, cougars, lions and tigers from zoos and circuses, indicating cross-infections between large felines.^34–38^ Eradication of *H. acinonychis* with antibiotic therapy resulted in disappearance of the gastric lesions, showing a causal connection of *H. acinonychis* infection and gastric disease.^39^ However, *H. acinonychis* strains do not contain the *cag*PAI, and the *vacA* gene is highly degenerated by mutations.^34,40^ Similar to *H. pylori*, *H. acinonychis* isolates express urease, flagellin, serine protease HtrA, neutrophil-activating protein NapA, lytic transglycosylase Slt, and γ-glutamyltranspeptidase GGT, but several outer membrane protein (OMP) coding genes are not expressed due to frameshift mutations, including *oipA*, *babB*, *hopQ*, *hopN*, *hopU*, and *sabA*.^38,40^

The next closest relatives of *H. pylori* are *Helicobacter cetorum* and *Helicobacter delphinicola*. All other *Helicobacter* species are genetically much more distinct (Figures 1(A,B)). *H. cetorum* was originally isolated from the main stomach of two wild, stranded Atlantic white-sided dolphins^41^ and later from the feces of wild Atlantic bottlenose dolphins^42^ and of captive animals, including a Pacific white-sided dolphin, an Atlantic bottlenose dolphin, and a beluga whale.^43,44^ Endoscopic examination of infected animals, which were reported to show lethargy and periodic regurgitation, revealed gastric inflammation and ulcers in the esophagus and the forestomach.^43^ Genome sequences of *H. cetorum* isolates from a dolphin and a beluga whale were 1.8 and 1.95 Mb in size, slightly larger than the genomes from *H. pylori*.^45^ These genomes showed that *H. cetorum* lacks the *cag*PAI and revealed divergent sets of OMP genes, the presence of the nickel-cofactored urease operon known from *H. pylori* plus an iron-cofactored urease also found in *H. felis* and *H. mustelae*, and several metabolic genes distinct from those in *H. pylori*. Moreover, the genome of the dolphin isolate MIT 99–5656 contained three additional *vacA* paralogs inserted five genes upstream of *vacA*.^45^ *H. delphinicola* was isolated from captive common bottlenose dolphins held at the Port of Nagoya Public Aquarium in Japan.^46^ Some of the animals were lethargic and showed signs of anorexia and dyspepsia. Endoscopy of the forestomachs and examination of gastric fluid samples revealed inflammation, bleeding, and ulcers.^46^

Infection with *H. pylori* is widespread among humans, approximately half of the global population carries this bacterium.^47^ An initial screening among wild Atlantic bottlenose dolphins revealed the presence of *H. cetorum* in about 50% of the examined dolphin population,^42^ suggesting that the prevalence of *H. cetorum* among dolphins might be as high as *H. pylori* among humans. However, besides the initial *H. cetorum* species description after the original isolation of the bacteria and the genome analysis of two isolates, very little is known about *H. cetorum*. Here, we performed a molecular characterization of four different *H. cetorum* isolates in comparison to four *H. pylori* laboratory strains. Based on the observed mild pathology in captive dolphins infected with *H. cetorum*,^41,43,44^ we were interested in comparing bacterial pathogenicity factors in *H. cetorum* and *H. pylori* and to evaluate their role in gastric disease-associated processes, including induction of inflammation, hemolysis, and damage of epithelial cells by vacuole formation and induction of DNA double-strand breaks (DSBs).

## Results

### Phylogenetic analysis

Gastric *Helicobacter* species colonize the stomach of their respective hosts.^48,49^ To analyze the relationship of *H. cetorum* with *H. pylori* and other gastric *Helicobacter* species, we generated a Neighbor-joining from their *16SrRNA* gene sequences using *Campylobacter concisus* as an outgroup (Figure 1(A)). In agreement with previous analyses,^2,43,45,49^ the gastric *Helicobacter* species clustered in two groups. The first cluster was composed of *H. salomonis*, *H. bizzozeronii*, *H. felis*, *H. heilmannii* and *H. suis*, and the second cluster contained *H. cetorum*, *H. delphinicola*, *H. pylori* and *H. acinonychis*. A Neighbor-joining tree based on concatenated housekeeping gene sequences (Figure 1(B)) confirmed the close relation of *H. cetorum*, *H. pylori* and *H. acinonychis*. Phylogenetic analyses of those sequences showed that the genetic diversity of the housekeeping genes (Figure 1(B)) was significantly larger in *H. cetorum* (Π_95_ = 4.94–9.19%) than in *H. pylori* (Π_95_ = 3.10–3.20%), indicating that *H. cetorum* is evolutionarily much older than *H. pylori*. Of all helicobacters, only those three species contained the *vacA* gene (Figure 1(C)). While present in full length in *H. cetorum* and *H. pylori*, *vacA* is present as 13 gene fragments and is thus highly degenerated in *H. acinonychis*.^34,40^ Figure 1(D) shows the *vacA* phylogeny of four *H. cetorum* isolates that were originally obtained from a Pacific white sided dolphin (MIT 01–5903), an Atlantic white sided dolphin (MIT 01–6096), and two Atlantic bottlenose dolphins (MIT 99–5656, MIT 01–6202), and from four selected human *H. pylori* strains (N6, G27, 60190 and P1).

#### *H.cetorum* electron microscopy analyses

**Figure 2.**
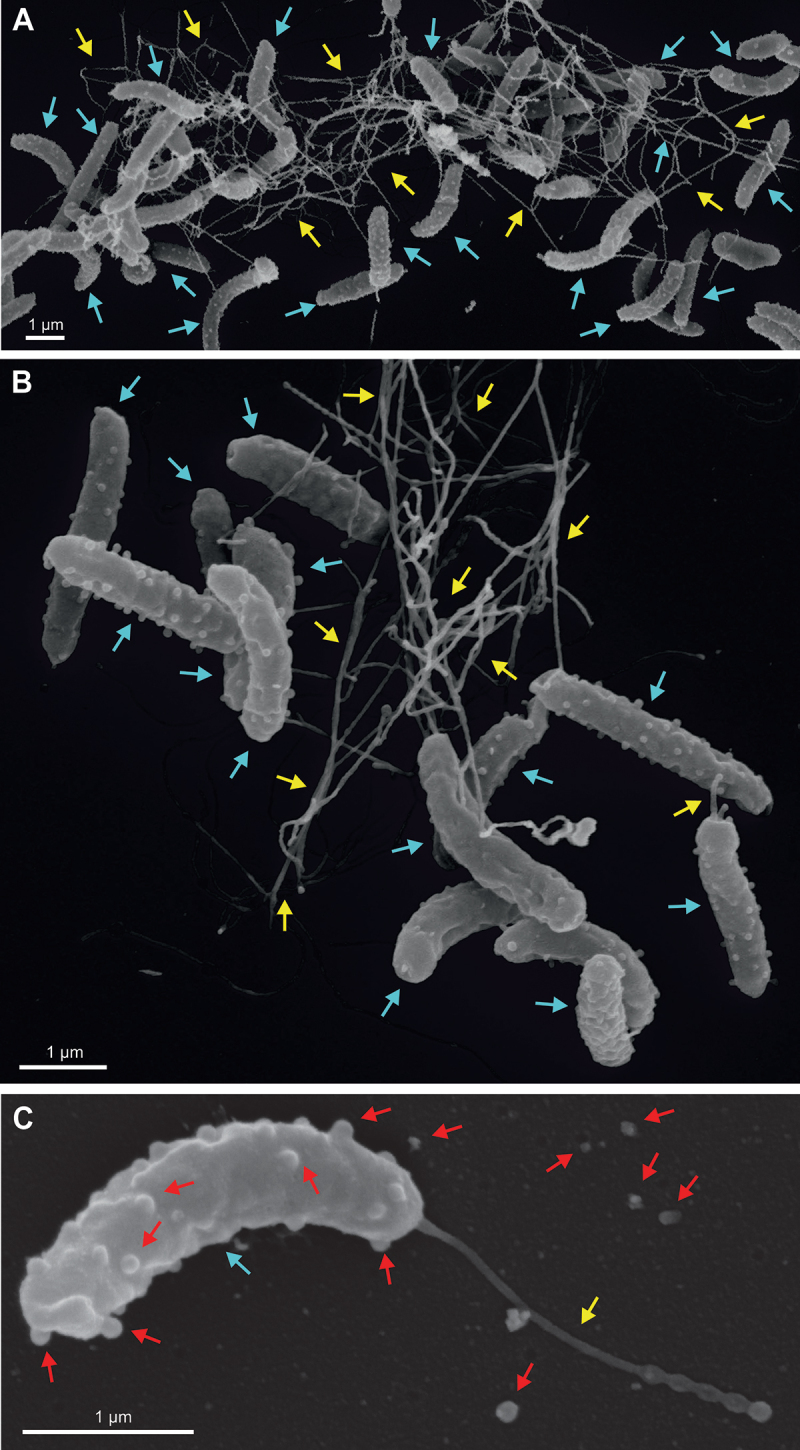
Scanning electron microscopy of *H. cetorum*. A) overview of multiple, curved *H. cetorum* strain MIT 01–5903 bacteria (blue arrows) with flagella (yellow arrows). B) enlarged view. C) image of a single bacterium (blue arrow) with a monopolar flagellum (yellow arrow) and multiple budding and released OMVs (red arrows). Representative pictures from two independent preparations are shown.

**Figure 3.**
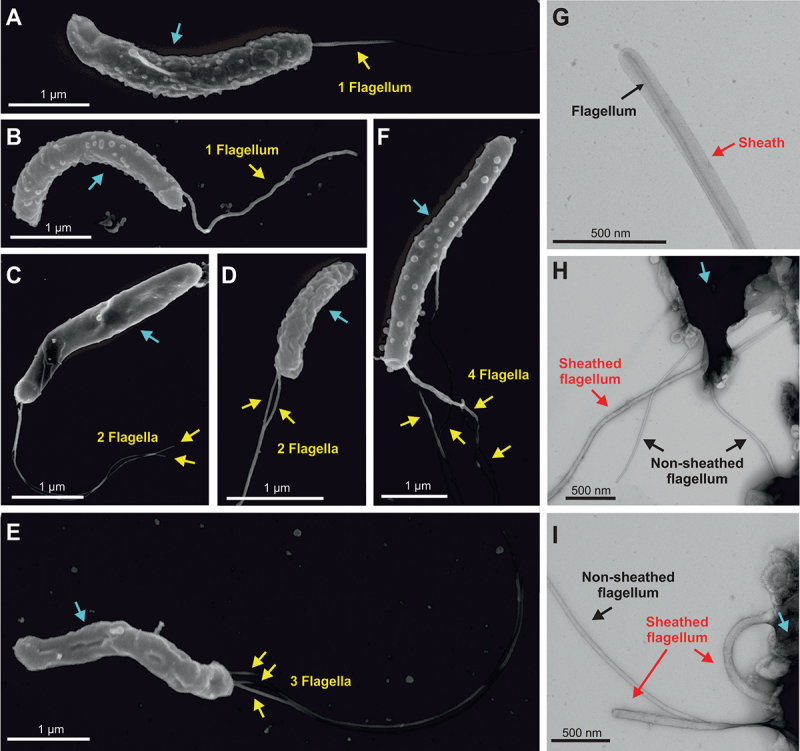
Morphological investigation of *H. cetorum* by scanning (A-E) and transmission electron microscopy (G-I). Single *H. cetorum* strain MIT 01–5903 and MIT 01–6096 bacteria (blue arrows) were analyzed, exhibiting either one monopolar flagellum (yellow arrows, panels A and B), two monopolar flagella (yellow arrows, panels C and D), three monopolar flagella (yellow arrows, panel E) or four monopolar flagella (yellow arrows, panel F). Negative contrast and transmission electron microscopy of the same *H. cetorum* samples (panels G-I) also revealed 1–4 monopolar flagella per bacterium (black arrows), some of which were covered with a sheath (red arrows). Representative pictures are shown from two independent preparations each.

All four *H. cetorum* isolates were analyzed by scanning electron microscopy (SEM) and transmission electron microscopy (TEM), yielding similar results. Representative pictures are shown from two strains, MIT 01–5903 and MIT 01–6096 (Figures 2, 3, S1 and S2). The SEM images showed typically curved, flagellated bacteria that are approximately 1.5–3 µm long and about 0.4 µm wide. The size and cell shape (curved or helical) varied slightly among the individual cells (Figure 2(A–C); Fig. S1A-B, Figure 3, blue arrows). Typical outer membrane vesicles (OMVs) are visible at the surface of many of these bacteria and in their immediate surroundings. These OMVs, which are initially formed at the bacterial surface and then secreted into the medium, are about 40–100 nm in diameter (Figure 2(C), red arrows), similar to OMVs from other Gram-negative bacteria. The bacteria that are usually cultured under microaerophilic conditions were very sensitive to oxidative stress. Exposure of *H. cetorum* to normal atmosphere for 10 min already resulted in the formation of coccoid bacteria (Fig. S2A-C, blue arrows). Similar coccoid forms of *H. pylori* are no longer cultivable and likely represent dead bacterial cells.^50^ In addition, *H. cetorum* expresses flagella that were up to 3–5 µm long. In contrast to *H. pylori* that usually forms a bundle of several mono-polar flagella,^11^ over 50% of the *H. cetorum* bacteria possessed a single polar flagellum (Figure 3(A,B) yellow arrows). About one-third of the bacterial cells had two polar flagella (Figures 3(C,D)), and the remaining cells had three (Figure 3(E)) or four flagella each (Figure 3(F)) or more. Thus, the number of flagella varied, even among individual bacteria from the same culture. Since the thickness of flagella in the SEM images seem to vary (e.g. in panels B and E), we additionally performed negative staining and TEM. The TEM images revealed the presence of 1–4 monopolar sheathed or non-sheathed flagella (Figure 3(G–F)), marked by red and black arrows, respectively. These sheaths had a diameter of about 35–50 nm compared to 20 nm of non-sheathed flagella.

#### *H.cetorum* strains express and secrete a functional urease

**Figure 4.**
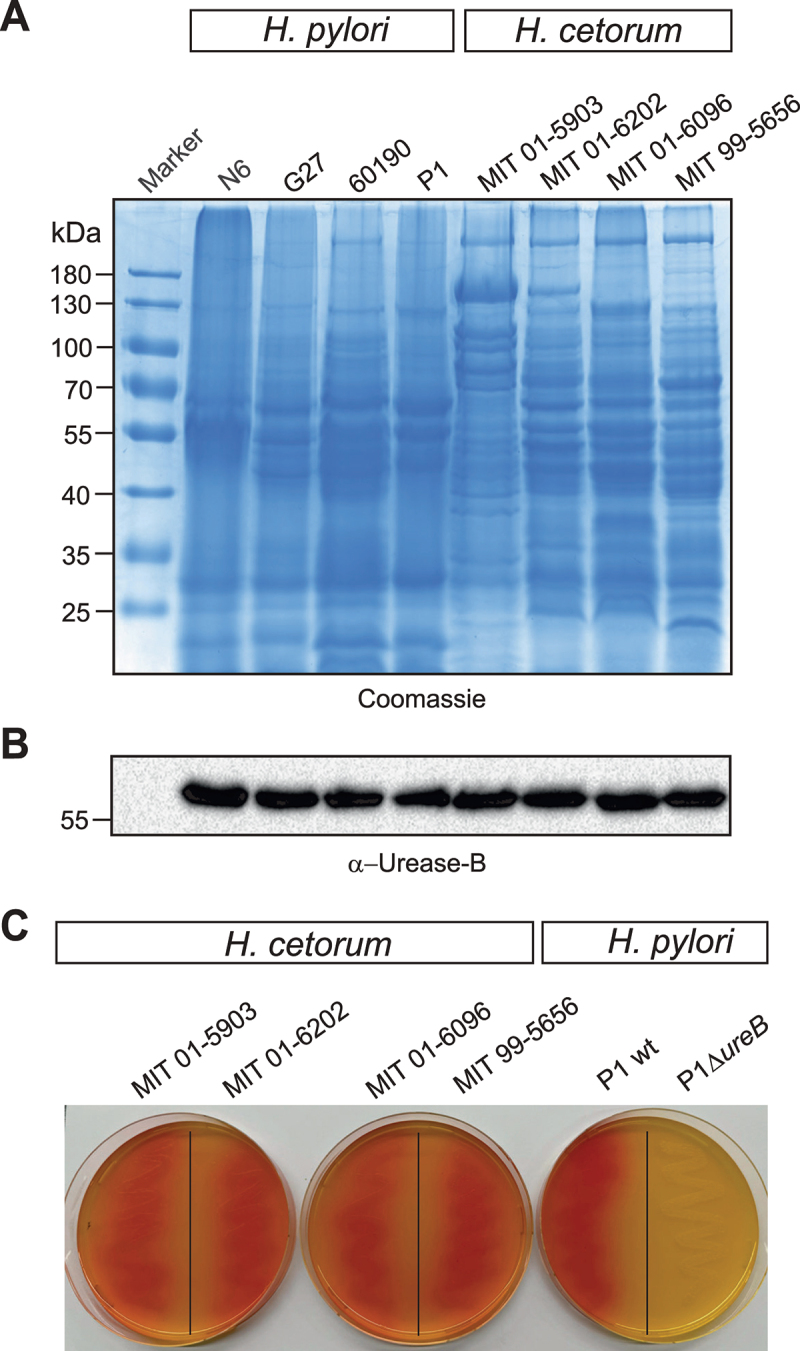
Protein profiles of *H. pylori* and *H. cetorum* and urease expression. A) Coomassie stain of total protein extracts of four *H. pylori* and four *H. cetorum* strains after separation by SDS-PAGE. B) Western blot with antibodies against the urease B subunit. C) positive urease test indicated by the red color during growth of four *H. cetorum* strains and *H. pylori* strain P1 on agar plates supplemented with urease. The isogenic *H. pylori* P1Δ*ureB* deletion mutant served as a negative control.

Coomassie-stained gels of total protein extracts revealed similar protein profiles among the four analyzed *H. pylori* strains, and showed only minor differences between the strains (Figure 4(A)). The protein patterns of the four *H. cetorum* strains were distinct from those of *H. pylori*. While the patterns from the three *H. cetorum* strains MIT 99–5656, MIT 01–6096, and MIT 01–6202 were relatively similar to each other, strain MIT 01–5903 had a different protein profile. A Western blot of the same protein extracts using an antibody specific for the *H. pylori* urease B protein (α-Urease-B) revealed a specific band at approximately 60 kDa for all samples, which indicated similar expression levels of urease by both *H. pylori* and *H. cetorum* (Figure 4(B)). Moreover, these data showed loading of similar total protein amounts and that the urease proteins are conserved (Fig. S3) to be recognized by the same antibody. In order to test whether the urease is secreted and active by *H. cetorum* similar to *H. pylori*, bacteria were cultured on acidified GC agar plates supplemented with urea, which is the substrate of the urease enzyme (Figure 4(C)). The observed red color change indicated comparative levels of urease secretion and activity by both species. An isogenic *H. pylori* urease deletion mutant included as control failed to induce the color change, as expected. In agreement with previously published data,^45^ the *H. cetorum* genomes of strains MIT 99–5656, MIT 01–6096 and MIT 01–6202 contained *ureA* and *ureB* genes coding for an iron-cofactored urease enzyme in addition to the 7-gene urease operon known from *H. pylori* (locus tags HP0067–HP0073 in *H. pylori* strain 26695) (Fig. S3). In strain MIT 01–5903, these extra urease genes were truncated and likely not functional.

#### *H.cetorum* is hemolytic against erythrocytes

**Figure 5.**
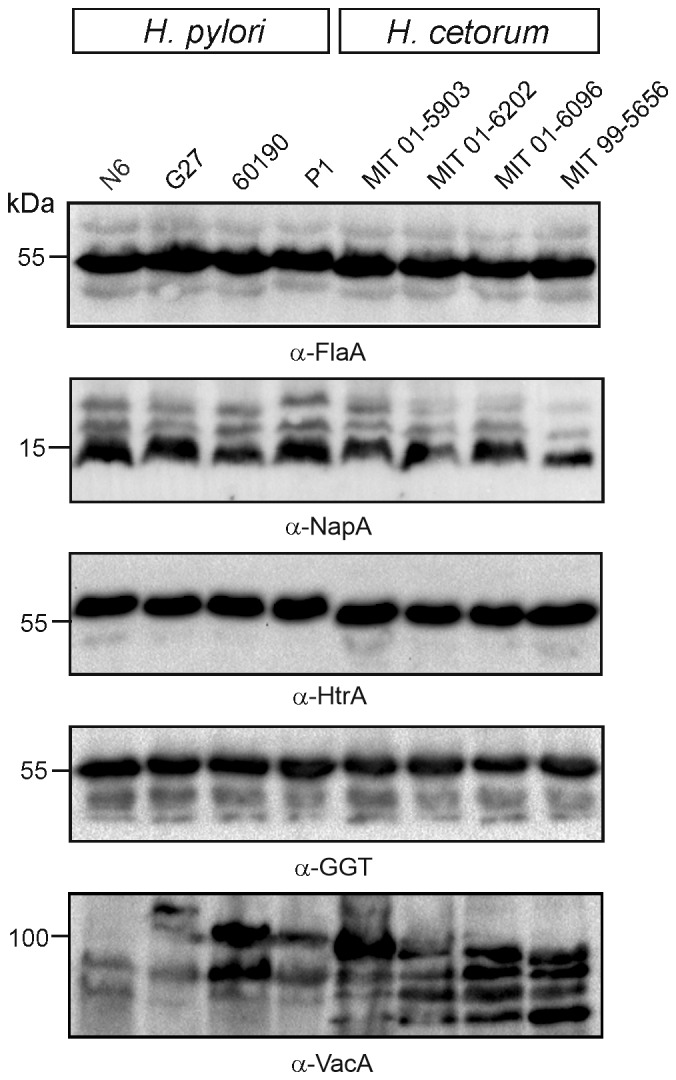
Western blots of SDS-PAGE separated cell extracts from *H. pylori* and *H. cetorum* strains. Antibodies against flagellin A, NapA, HtrA and GGT showed similar protein patterns between *H. pylori* and *H. cetorum*. Hybridization with α-VacA antibody revealed additional, smaller bands in *H. cetorum* VacA.

**Figure 6.**
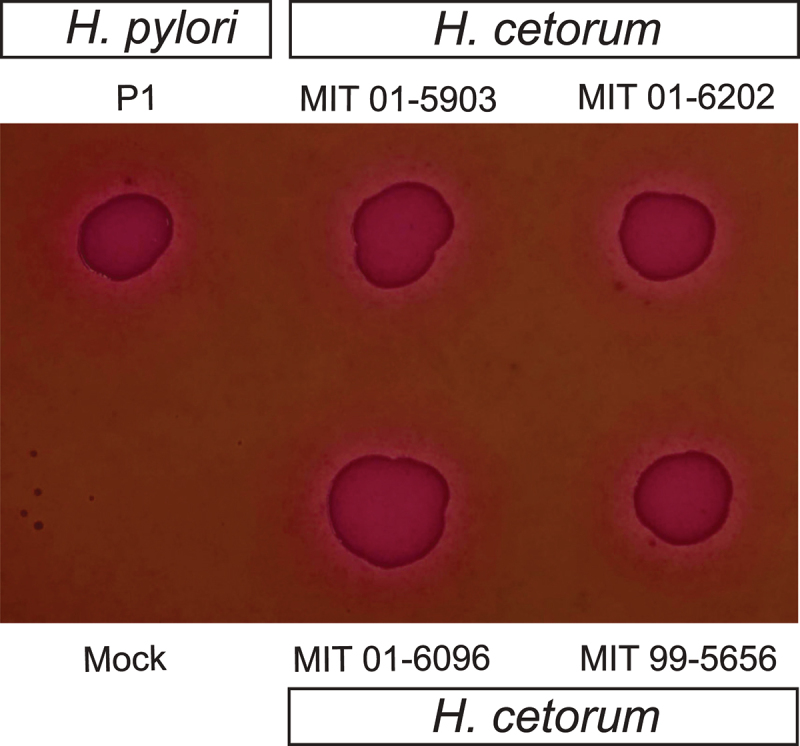
Blood hemolysis. Hemolysis caused by indicated *H. pylori* and *H. cetorum* strains grown for 3 days on blood agar plates.

Other virulence factors conserved in both *H. pylori* and *H. cetorum* were also analyzed for protein expression during infection (Figure 5). Western blots showed expression of the flagella protein flagellin A, neutrophil-activating protein NapA, serine protease HtrA, γ-glutamyl transpeptidase GGT, and vacuolating cytotoxin VacA. Alignments of these proteins are shown in Figs. S4-S8. Next, the strains were analyzed for their ability to trigger hemolysis of red blood cells embedded in agar plates as *H. pylori* is known to display high hemolytic activity for iron acquisition from the host.^51^ Both *H. pylori* and *H. cetorum* exhibited strong hemolytic activity toward erythrocytes during incubation on blood agar (Figure 6), indicating that *H. cetorum* has a similarly high hemolytic activity as *H. pylori*, which is consistent with the large number of iron acquisition genes in the genomes of both species.

#### *H.cetorum* VacA induces cell vacuole formation similar to *H.pylori* s1/m2-type strains

**Figure 7.**
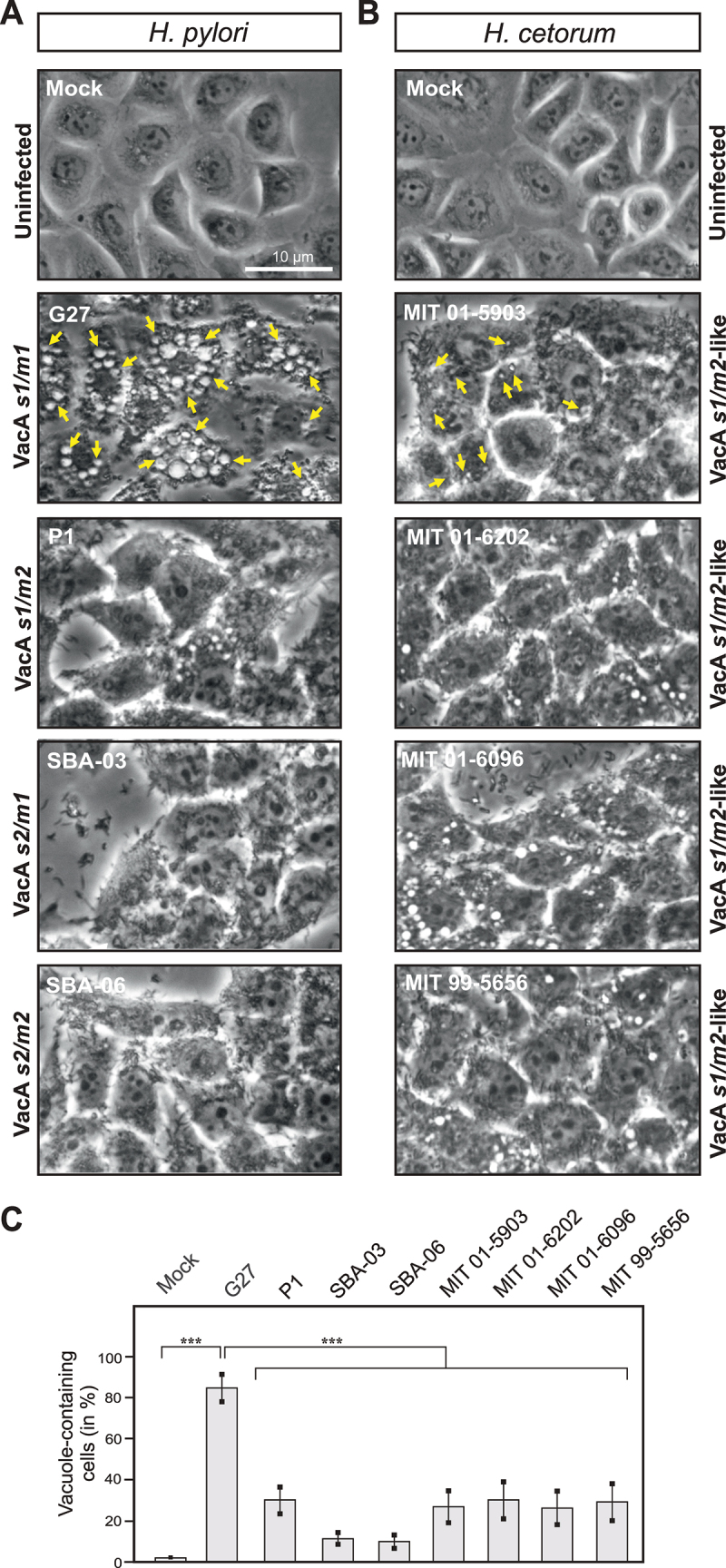
Vacuolization of epithelial cells during infection with *H. pylori* and *H. cetorum*. A) degree of vacuole formation during infection with VacA *s1/m1* (G27), *s1/m2* (P1), *s2/m1* (SBA-03), and *s2/m2* (SBA06). B) vacuole formation caused by *H. cetorum* was comparable to *s1/m2* from *H. pylori* and termed *s1/m2*-like. Intracellular vacuoles are exemplarily marked with yellow arrows in two pictures (infection with G27 and MIT 01–5903). C) quantification of vacuolization.

Since the *vacA* gene is only present in *H. pylori* and *H. cetorum*, and is heavily degenerated in *H. acinonychis*, the evolutionary origin of *vacA* is unclear. Therefore, we compared the VacA cytotoxin from both species in greater detail. Analysis of VacA in Western blots revealed the same bands in both species (at about 98 kDa and 88 kDa), but also the presence of additional, smaller fragments (ca. 85 kDa and 80 kDa) in *H. cetorum* VacA compared to *H. pylori* VacA (Figure 5, bottom). This suggests that *H. cetorum* VacA may be further processed compared to the p95 and p88 fragments known from *H. pylori*. As next, all four *H. cetorum* strains were tested for their ability to induce the formation of vacuoles during infection of the human gastric epithelial cell line AGS (Figure 7). For comparison, four *H. pylori* strains were selected that differ in their respective VacA alleles: G27 with the highly cytotoxic allele combination *s1/m1*, strain P1 with the moderately cytotoxic alleles *s1/m2*, the recently isolated strain SBA-03 containing the unusual and very rare allele combination *s2/m1*, and the non-cytotoxic *s2/m2* strain SBA-06.^52^ As expected, the *s1/m1*-type strain G27 triggered strong formation of vacuoles in the epithelial cells, and less pronounced host cell vacuolization was observed for the *s1/m2*-type strain P1. In contrast, cell vacuolization was nearly not observed in cells infected with *s2/m1-* or *s2/m2*-type *H. pylori* (Figure 7(A)). In addition to *H. pylori*, cell vacuolization was also induced by *H. cetorum*. By comparison, *H. cetorum*-triggered vacuole formation in host cells was less pronounced than vacuolization during infection with *H. pylori s1/m1*-type strains (Figure 7(B)). Visual inspection of the images (Figure 7(B)) as well as quantification of the number of vacuole-containing cells (Figure 7(C)) revealed that *H. cetorum*-induced vacuole formation was comparable to infection with *H. pylori s1/m2*-type strains. Therefore, we propose to designate VacA-triggered vacuolization by *H. cetorum* as *s1/m2*-like (Figure 7(B)).

**Figure 8.**
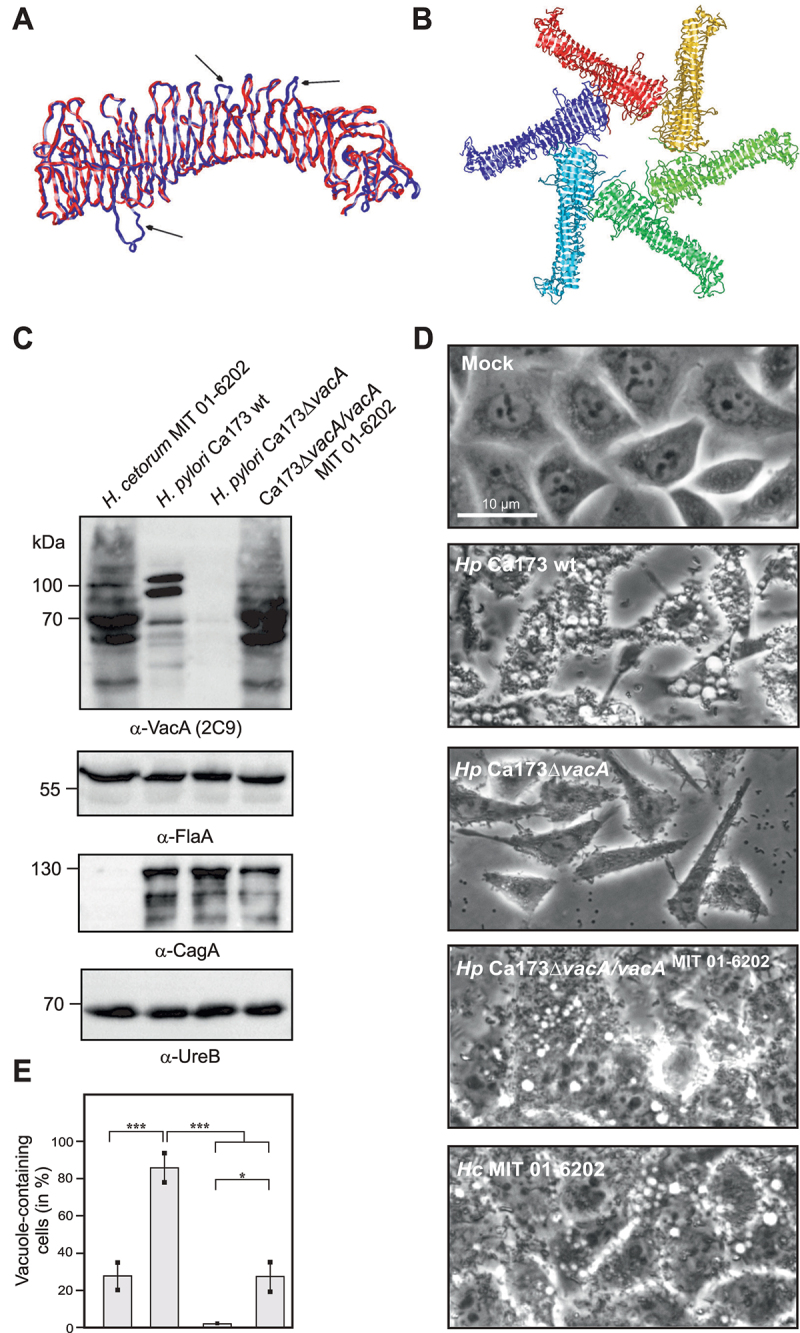
VacA 3D-structure and function. A) computer model of *H. cetorum* VacA (blue) superimposed on the experimental 3D-structure of *H. pylori* VacA (red). Black arrows point to protruding loops of VacA from *H. cetorum* compared to *H. pylori*. B) model of the *H. cetorum* VacA hexamer with the individual subunits shown in different colors. C) complementation of *vacA* in *H. pylori* strain Ca173 with the *vacA* gene from *H. cetorum* shown by Western blot against VacA. Western blots against FlaA, CagA and UreB served as loading controls. D) vacuolization of host cells caused by *H. pylori* Ca173, an isogenic Ca173Δ*vacA* deletion mutant, the deletion mutant complemented with *H. cetorum vacA*, and the corresponding *H. cetorum* wt strain MIT01–6202. E) quantification of vacuole formation.

Alignment of *H. pylori* and *H. cetorum* VacA sequences showed long protein stretches with high homology (Fig. S8). In addition, computer modeling predicted the VacA 3D-structures to be very similar (Figure 8(A)), with most amino acid stretches overlapping between *H. pylori* (red) and *H. cetorum* (blue) proteins. Just like the *H. pylori* toxin, the hexameric *H. cetorum* VacA was predicted to exhibit a topology in which the subunits are arranged around a central pore (Figure 8(B)). However, there were slight differences in the form of some *H. cetorum* VacA loops protruding from the *H. cetorum* VacA structure (Figure 8). Those protruding loops are likely accessible to proteases, which might explain the presence of at least two additional bands in the Western blots that are somewhat smaller in size than the p88 and p95 fragments observed from *H. pylori* VacA (Figure 5). To investigate *H. cetorum* VacA in more detail, the *vacA* gene from *H. pylori* strain Ca173 was complemented with *vacA* from *H. cetorum* strain MIT 01–6202. Western blots confirmed *H. cetorum* VacA re-expression in the *H. pylori* strain background (Figure 8(C)). Western blots of flagellin, CagA, and urease served as internal controls. Subsequently, the complemented mutant was assessed for formation of host cell vacuoles during infection of AGS cells. Phase contrast microscopy confirmed vacuole formation by the complemented mutant (Figure 8(D)). The images showed that vacuole forming was similar to that induced by *H. cetorum* wt bacteria (Figure 8(D,E)).

#### *H.cetorum* fails to induce NF-κB response and IL-8 secretion

**Figure 9.**
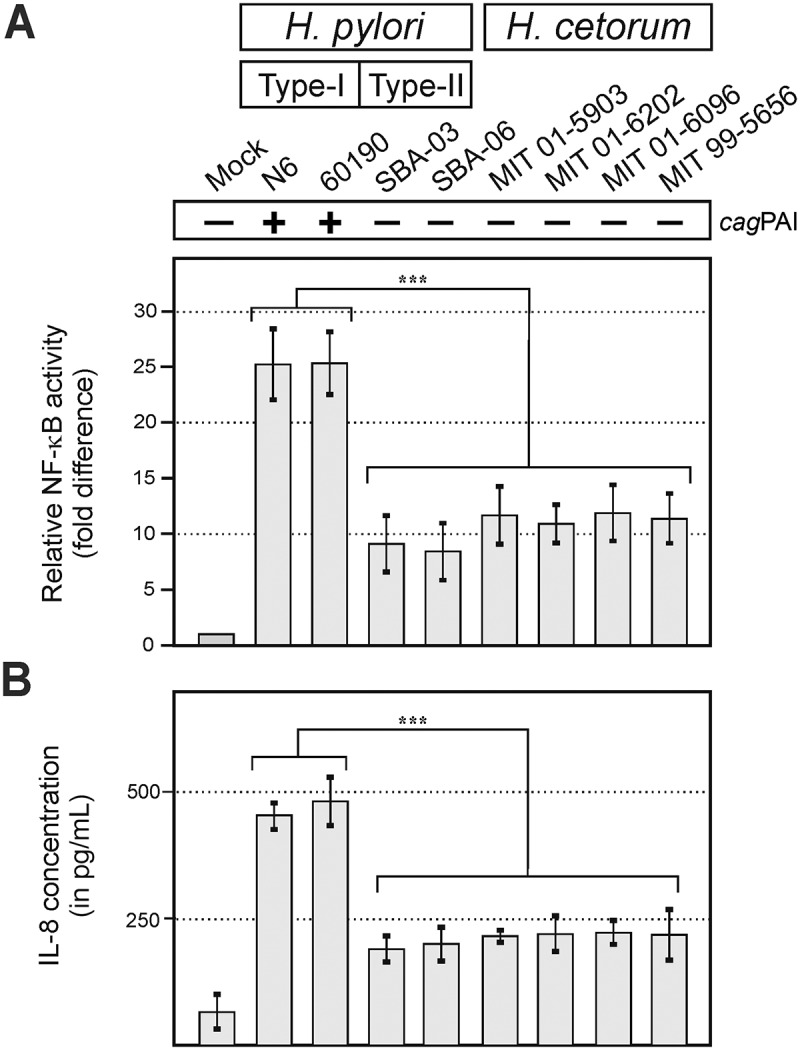
Pro-inflammatory host response during infection of epithelial cells by *H. pylori* and *H. cetorum*. A) activation of NF-κB relative to uninfected cells (mock) and (B) secretion of IL-8 into the supernatant. *H. cetorum* failed to induce a pronounced inflammatory response compared to *H. pylori*.

*H. pylori* infection is known to trigger an inflammatory host response that involves activation of transcription factor NF-κB and subsequent secretion of inflammatory cytokines such as IL-8.^19^ Whether infection with *H. cetorum* induces a similar host inflammation response is unknown. Therefore, AGS gastric epithelial cells infected with either *H. pylori* or *H. cetorum* bacteria were assessed for activation of NF-κB using a Quanti-Blue reporter assay, and the amounts of secreted cytokines were determined by ELISA against IL-8 (Figure 9). Quantification of the data revealed strong activation of NF-κB (Figure 9(A)) and IL-8 expression (Figure 9(B)) by *H. pylori*, but only very weak, if any, activation of either of those inflammation markers by infection with *H. cetorum*.

#### *H.cetorum* bacteria do not or only moderately activate toll-like receptors

**Figure 10.**
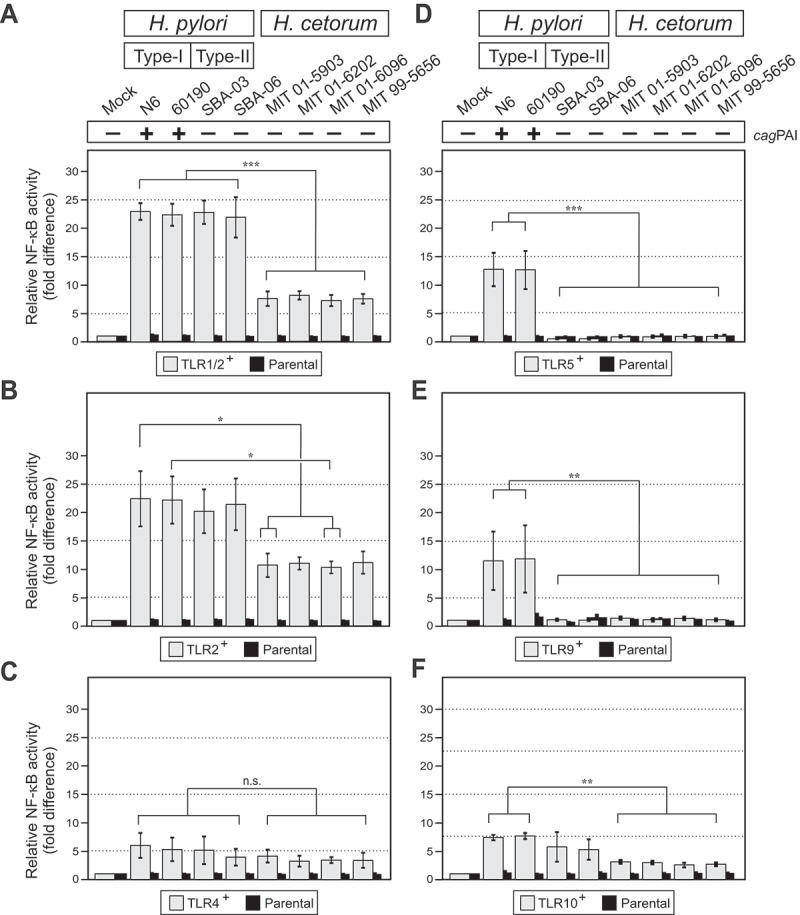
Recognition of *H. pylori* and *H. cetorum* by host cell TLRs. A-F) infection of HEK cells expressing the individual TLRs with *H. pylori* induced between 4-fold and 20-fold higher NF-κB reporter activity compared to the uninfected mock control. *H. cetorum* was not recognized by TLR4 (C), TLR5 (D) and TLR9 (E), and triggered only moderate activation of NF-κB through TLR1/2 (A), TLR2 (B) and TLR10 (F).

In addition, *H. pylori* is known to be recognized by a wide range of toll-like receptors (TLR1, TLR2, TLR4, TLR5, TLR9, and TLR10)^53^^,^^54^, but whether *H. cetorum* is also recognized by any of those TLRs is unknown. Therefore, the activation of TLRs was analyzed during exposure of Hek293 TLR reporter cells to *H. cetorum* in comparison to exposure to *H. pylori* (Figure 10). As expected, infection with *cag*PAI-positive *H. pylori* strains (N6, 60190) activated all analyzed TLRs, and increased the reporter activity relative to the uninfected and parental controls by approximately 22-fold (TLR1/2), 20-fold (TLR2), 4-fold (TLR4), 12-fold (TLR5), 11-fold (TLR9) and 6-fold (TLR10). The two *cag*PAI-negative *H. pylori* strains SBA-03 and SBA-06,^52^ which were included as controls, stimulated TLR1/2, TLR2, TLR4, and TLR10 similar to *cag*PAI-positive *H. pylori*. However, in agreement with previous data^55–57^, *cag*PAI-deficient *H. pylori* cannot activate TLR5 and TLR9. In contrast, *H. cetorum* were not recognized by TLRs (TLR4, TLR5, TLR9) or activated TLRs only moderately, one-third to half as strong as *H. pylori* (TLR1/2, TLR2, TLR10). Together, those data show that exposure of epithelial cells to *H. cetorum* activates only a few TLRs and causes a significantly weaker inflammatory response than *H. pylori*.

#### *H.cetorum* does not induce DNA double-strand breaks (DSBs) in host cell chromosomal DNA

**Figure 11.**
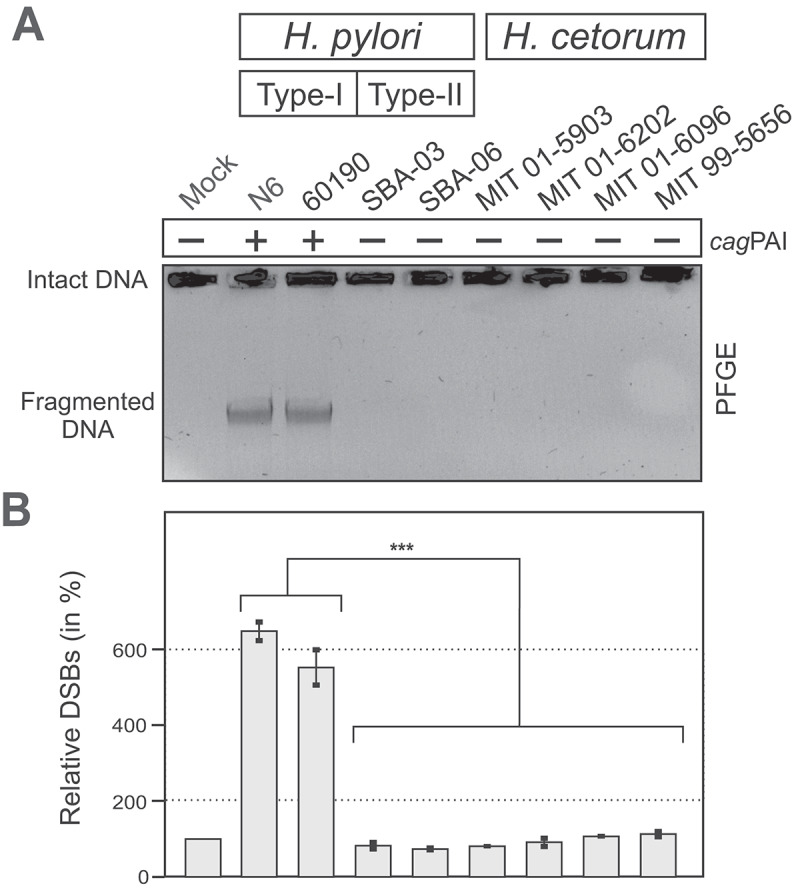
Induction of DNA double-strand breaks during infection with *cag*PAI-positive *H. pylori*, but not *cag*PAI-negative isolates or *H. cetorum*. (A) PFGE-analysis of host chromosome damage. (B) DNA fragmentation (bottom) was normalized against intact chromosomal DNA retained in the loading well (top), and against the uninfected control (lane 1) that was set to 100%. All data are represented as mean ± SEM. *p* < 0.001 (***) was considered statistically significant.

Finally, various earlier reports have shown that *H. pylori* infection induces substantial DNA damage in the host chromosome in a *cag*PAI-dependent fashion.^58–61^ This is triggered through the introduction of DSBs that arise during induced transcription in RNA/DNA-hybrid structures (R-loops) upon ADPH-stimulated NF-κB activation, followed by the onset of DNA repair processes.^61^ These DSBs result in genomic fragility and accumulation of DNA mutations, which enhance cancerogenic gene modifications in the host. Pulsed-field gel electrophoresis (PFGE) revealed strong fragmentation of host chromosomal DNA upon infection with *cag*PAI-positive *H. pylori* isolates, but not with *cag*PAI-negative *H. pylori* and *H. cetorum* strains (Figure 11).

## Discussion

*Helicobacters* are known to comprise enterohepatic species that inhabit the intestinal tract and/or the gall bladder, and gastric species that colonize the stomach of their respective hosts.^2^ While there is a small possibility that a species more closely related to the human stomach bacterium *H. pylori* exists, the closest currently known relatives are *H. acinonychis* from large felines and *H. cetorum* and *H. delphinicola* from dolphins (Figure 1(A)). Genome comparisons showed that *H. acinonychis* arose in Southern Africa by a host jump of *H. pylori* of the biogeographic population hpAfrica2 from humans to large cats likely when an *H. pylori*-infected human was eaten by a large feline. This jump between hosts, which happened approximately 100,000 years ago, indicates that *H. acinonychis* directly descendent from *H. pylori*.^40^ Colonization of lions, cheetahs, cougars, and tigers with *H. acinonychis* indicated that cross-infection occurred later among other big cats that were kept together in captivity.^35–38^ An analysis of pathogenicity-related factors revealed *H. acinonychis* expression of flagellin, urease, serine protease HtrA, neutrophil activating protein NapA, γ-glutamyl transpeptidase, and lytic transglycosylase Slt.^38^ In contrast, the *cag*PAI, the *cagA* gene and the *babA* gene were absent from the genome, and other *H. pylori* virulence factors such as VacA and OipA were not expressed because of several frameshift mutations.^34,40^

The next closest known relatives of *H. pylori* are *H. cetorum* and *H. delphinicola* (Figure 1(A)). *H. cetorum* has been isolated from several species of wild and captive dolphins and from a beluga whale held in the Mystic Aquarium in Connecticut, USA.^41–44^ Whether wild whales also carry *H. cetorum* is questionable because *H. cetorum* infection of the beluga whale had likely occurred in captivity. This particular animal was reported to have shared an enclosed basin with a bottlenose dolphin in which gastric ulcers were detected during a health checkup.^44^ Thus, similar to *H. acinonychis* among large felines in zoos and circuses, cross-infection of *H. cetorum* appears to occur among captive cetaceans. *H. delphinicola* was first identified in bottlenose dolphins at the Nagoya Public Aquarium, Japan, and subsequently found among dolphins in several animal facilities in Japan.^46^ Apparently, simultaneous infection with both *H. delphinicola* and *H. cetorum* seems frequent in these facilities, as 79 of the 82 dolphins tested were positive for *H. cetorum* and 45 were positive for *H. delphinicola*.^57^ However, we propose that *H. delphinicola* might simply represent a different phylogenetic clade of *H. cetorum*, similar to the hpAfrica2 population of *H. pylori* that is distinct from other *H. pylori* groups (Figure 1(A)).^1,2^ In agreement, a BLAST nucleotide search of the published *H. delphinicola 16SrRNA* gene sequence yielded hits for both *H. delphinicola* and *H. cetorum* with 100% sequence identity, with *H. cetorum* isolated from a common dolphin (*Delphinus delphis*) at the southwest coast of England (Genbank accession FN565165). Unfortunately, there is currently no publically available genome for *H. delphinicola* that would allow a detailed analysis. Future studies should clarify this question.

Histological examination of stomach mucosa samples from stranded, *H. cetorum*-infected dolphins showed gastric lesions, including signs of mild multifocal gastritis.^41^ Likewise, captive animals with clinical signs such as vomiting and inappetence were shown to have ulcers in their forestomachs,^44,46^ and histology images of the main stomach revealed mild mucosal erosion with mononuclear cell infiltrates in the lamina propria.^43^ Immediately adjacent to those areas of gastritis, including pseudolymphoid follicles in the main glandular portion of the stomach, spiral bacteria were found on the gastric epithelium. Given that such lymphoid follicles are often seen in glandular stomach of *Helicobacter* infection in mice, this suggested a causal relationship between *H. cetorum* and the observed pathological changes.^43^ Besides the initial species description, including the relatively mild pathology, and the description of two *H. cetorum* genomes isolated from a dolphin and the beluga whale, very little was known about *H. cetorum*. Therefore, we analyzed four *H. cetorum* isolates in comparison to *H. pylori* strains. In agreement with previous data,^43^ electron microscopy images of *H. cetorum* showed flagellated curved bacteria that were approximately 1.5–3 by 0.4 µm in size. Our scanning electron microscopy images taken from over 600 cells from each of the four analyzed strains showed bacteria with one, two, three, and even four flagella protruding from one end of the bacterial cell (Figures 2, 3). Thus, all bacteria had mono-polar flagella, which is in contrast to a previous transmission electron micrograph of *H. cetorum* strain MIT99–5656 that showed bipolar flagella with a single flagellum at each cell end.^43^ Just like other *Helicobacter* bacteria, *H. cetorum* was shown to apply a run-reverse-reorient mechanism for its motility in liquid and semi-solid media, and also in gastric mucin.^62^ Moreover, our results revealed that at least part of the *H. cetorum* flagella was sheathed, similar to flagella reported from *H. pylori*^63^ and *H. acinonychis*.^38^ Indeed, the gene encoding the flagella sheath protein HpaA was identified in the genomes of all three species.

Similar to *H. pylori* and *H. acinonychis*, *H. cetorum* expresses and secretes urease to buffer the acidic surrounding in the stomach (Figure 4). In this regard, urease expression appears to be a common trait as numerous gastric *Helicobacter* species secrete nickel-cofactored urease to buffer the acidic pH.^64,65^ A tblastn search against the NCBI RefSeq Genome Database revealed that, in addition to *H. pylori*, *H. acinonychis* and *H. cetorum*, those include *H. bizzozeronii*, *H. salomonis*, *H. felis*, *H. heilmannii*, *H. vulpis*, *H. labacensis*, *H. mehlei* and *H. suis*¸ and even several enterohepatic species such as *H. mustelae* and *H. hepaticus*. In addition, *H. cetorum* possesses the *ureA*/*ureB*-encoded iron-cofactored urease also present in *H. mustelae*, *H. felis*, *H. salomonis*, *H. cynogastricus*, *H. baculiformis* and *H. acinonychis*. Recently, both urease gene clusters were also found in a set of *H. pylori* isolates from indigenous Siberians and native Americans.^8,66^

Functional VacA is only expressed by *H. pylori* and *H. cetorum*. In addition, *H. acinonychis* possesses a highly degenerated gene with multiple frameshifts.^40,45^ Since VacA is not present in other species, including any other gastric and enterohepatic helicobacters, the origin of VacA is an enigma. Therefore, we looked at VacA in more detail and compared the predicted structure of *H. cetorum* VacA to the published cryo-EM structure of *H. pylori* VacA.^67^ The VacA monomers as well as the hexameric pore-forming toxins were very similar (Figure 8(A,B)), but several *H. cetorum* VacA loops stuck out from the protein overlay. We hypothesize that those protruding loops can be accessed by proteases because Western blots showed two smaller bands in the *H. cetorum* protein extracts compared to *H. pylori* (Figures 5, 8(C)). Given that these smaller extra bands were present in all four analyzed *H. cetorum* samples, this suggests additional cleavage sites in VacA rather than post-translational processing of VacA encoded by the three additional *vacA* paralogs found in the genome of strain MIT 99–5656, because those paralogous gene copies are only present in strain MIT 99–5656, but not in any of the other three *H. cetorum* genomes. Interestingly, two of those paralogs (*vacA2* and *vacA3*) were also found in several of the above mentioned *H. pylori* strains isolated from indigenous Siberians and native Americans.^66^ In addition, epithelial cells infected with *H. cetorum* exhibited vacuolization that was comparable to cells infected with *H. pylori* containing *s1/m2*-type VacA (Figures 7, 8(D)). Thus, this *s1/m2*-like VacA from *H. cetorum* triggered a relatively mild vacuolization. In addition, both species were hemolytic against erythrocytes, as indicated by halos surrounding the bacteria on blood agar plates (Figure 6). While *H. pylori* was known to sequester iron from host cells for its metabolism,^68,69^ which can lead to iron deficiency anemia diseases in children and adults,^70,71^ it is unclear whether iron acquisition by *H. cetorum* similarly affects the health of dolphins. Taken together, the two species are not only evolutionarily closely related, but also display a similarly high urease (Figure 4), hemolytic (Figure 6), and VacA activity (Figures 5, 7, 8).

In contrast to *H. pylori*, incubation of epithelial cells with *H. cetorum* induced hardly any inflammatory response in cultured gastric epithelial cells (Figure 9), which is probably associated with the absence of the *cag*PAI-encoded T4SS and *cagA* oncogene in *H. cetorum*,^45^ as well as with the inferior recognition by TLRs in reporter cells (Figure 10). TLR4, TLR5, and TLR9 failed to detect *H. cetorum* altogether, and recognition by TLR1, TLR2 and TLR10 was poor. While stimulation of a TLR2-based reporter system was about 10-fold compared to the baseline, it was still only half of that induced by *H. pylori* (Figure 10). Likewise, cancer-associated DSBs in host chromosomal DNA were strongly pronounced during infection with *cag*PAI-positive *H. pylori* strains, but not *cag*PAI-negative *H. pylori* and *H. cetorum* isolates (Figure 11). Taken together, incubation of epithelial cells with *H. cetorum* induced a very mild overall reaction. This leads to the hypothesis that *H. cetorum* represents a commensal or only moderately pathogenic bacterium in the stomach of dolphins that is comparable to the significantly less pathogenic *cag*PAI-negative (also known as type II) *H. pylori* strains in humans. However, we note that all infection experiments were performed with the human gastric epithelial cell line AGS. Human cells may differ from dolphin epithelial cells in terms of bacterial adherence and overall pathogen-host interaction, which may have contributed to the observed low pathogenicity, particularly given the reported ulcers and gastritis in dolphins.^41,43,46^

Given that *H. cetorum* is evolutionarily much older than *H. pylori*, we propose that *H. pylori* may have originated by a host jump of an *H. cetorum*-like ancestor from dolphins to early humans. Hypothetically, early humans ate stranded *H. cetorum*-infected dolphins, and thus contracted the bacteria. Subsequently, the bacteria adapted to the new host and eventually evolved into *H. pylori*, which was followed by spread of the bacteria in the human population. This scenario would be similar to the origin of *H. acinonychis* that supposedly arose from a host jump of *H. pylori* from early humans to large cats after *H. pylori*-infected humans were eaten by lions.^40,72^ When did the proposed host jump of *H. cetorum* from dolphins to humans happen? Based on mitochondrial (mt) DNA analyses human matrilineal diversity coalesces to a “mitochondrial Eve” approximately 200,000 years ago when mtDNA haplogroups L1-L6 split from haplogroup L0 that is associated with the former hunter-gatherer people in southern Africa known as the Khoi and San (Khoisan).^73,74^ At the same time, the *H. pylori* phylogeny split into two major lineages: 1) the population hpAfrica2 from southern Africa, which is characteristic for the Khoisan, and 2) all other biogeographic *H. pylori* populations in Africa and elsewhere in the world.^2^ On average, housekeeping gene sequences from *H. pylori* of the hpAfrica2 population show a genetic distance of 0.049 to *H. pylori* from any other population (Figure 1(B)). The average genetic distance of *H. pylori* to *H. cetorum* is 0.1527, and thus roughly three times as large (Figure 1(B)). Given the 200,000-year coalescence estimate for *H. pylori*, this results in a time of the most recent common ancestor (TMRCA), and thus, split of *H. cetorum* and *H. pylori* approximately 623,000 years ago. This coalescence date is in agreement with a previous study,^49^ in which the divergence between *H. pylori* and *H. cetorum* was calculated to be 610,000 years ago.

*H*. *pylori* contains the *cag*PAI, but *H. cetorum* does not, indicating that *H. pylori* must have acquired it after the divergence from *H. cetorum*. In addition, the average GC content of the *cag*PAI genes (35%) is lower than the GC content of the rest of the *H. pylori* genome (39%), which suggests lateral (from another bacterium) or horizontal (from archaea or eukaryotes) gene transfer into *H. pylori*.^75^ However, the evolutionary source of the *cag*PAI import is still unknown. The *cag*PAI is not present in all biogeographic *H. pylori* populations.^20,76,77^ In Africa, the *cag*PAI is entirely missing in the hpAfrica2 population, but is present in all hpAfrica1 strains, and is variably present in strains of the hpNEAfrica population (Figure 1(B)). Outside of Africa, the *cag*PAI is (variably) present in all *H. pylori* populations. This indicates that the *cag*PAI was imported in Africa after the divergence of the hpAfrica2 population from all other *H. pylori* that was estimated to 200,000 years ago.^2^ But the acquisition of *cag*PAI happened before the Out-of-Africa migration that was estimated to having occurred approximately 60,000 years ago,^1,78^ and thus between 200,000 and 60,000 years ago.

Taken together, in this study we have performed a detailed molecular characterization of four different *H. cetorum* strains from dolphins compared to *H. pylori* laboratory strains. Since *H. cetorum* is evolutionarily older than *H. pylori*, we propose that *H. cetorum* represents a direct ancestor of *H. pylori* that arose after a host jump over 623,000 years ago, which is the time of the coalescence of the two species. Remarkably, both *H. pylori* and *H. cetorum* express a highly active urease and trigger hemolysis, which is in agreement with the assumption that both bacteria must counteract the acid environment in the stomach and must acquire iron for their metabolism. However, *H. cetorum* induced only weak VacA-dependent vacuole formation and no visible DNA damage compared to highly virulent *H. pylori*. In addition, *H. cetorum* activated only a few TLRs and caused significantly weaker pro-inflammatory responses than *H. pylori*, suggesting that *H. cetorum* is a commensal or an only moderately pathogenic bacterium in the stomach of dolphins, comparable to the less pathogenic *cag*PAI-negative *H. pylori* strains in humans. These new studies are in well agreement with the mild pathology observed in captive dolphins infected with *H. cetorum* as discussed above.^41,43,46^ Future studies should inaugurate the genetics in *H. cetorum* for in depth analyses of the virulence factors, combined with establishing a suitable animal model system for *in vivo* infection studies.

## Materials and methods

### Bacterial culture

*H. pylori* laboratory wt strains N6, G27, 60190, P1, and Ca173 (*cag*PAI-positive), and the recently isolated SBA-03 and SBA-06 (*cag*PAI-negative) were described.^15,21,52,55,56^ The urease mutant was generated as reported previously.^79^ All *H. pylori* variants were cultured on GC agar plates supplemented with 10% horse serum, 1% vitamin mix and antibiotics and incubated at 37°C for 7 days in a microaerophilic atmosphere generated by CampyGen gas-generation sachets (Oxoid) in anaerobic jars.^80^ *H. cetorum* strains MIT99–5656, MIT01–5903, MIT01–6202^43^ and MIT01–6096^42^ were grown on BHI blood agar plates under microaerophilic conditions as described.^45^ For the urease test, agar culture plates containing 600 μg/mL urea and 100 μg/mL phenol red were acidified to pH 5 using 1 M HCl.^38^ For the analysis of hemolytic activity, the bacteria were grown on Colombia blood agar plates (Fisher Scientific, Cat# 10463833). Hemolysis, i.e. the formation of a pink zone around the culture, which indicates bacteria-induced hemolysis of erythrocytes in the culture medium, was assessed by visual inspection. Chromosomal DNA was isolated using the Gene Jet DNA Purification Kit (Invitrogen) following the protocol of the supplier.

### Genetic analyses

*16SrRNA* and housekeeping genes were downloaded from the Bacterial Isolate Genome Sequence database (BIGSdb) hosted at the pubmlst.org website,^81^ or were extracted from genome accessions (JAHGXX000000000, JAXTOX000000000, VAPN00000000, CP001173, NC_017735, FZMW00000000, FZMU00000000, FZMR00000000). For validation, a 1.2 kb DNA fragment of the *16SrRNA* gene was PCR amplified and sequenced from the four *H. cetorum* strains as described.^38^ The 16SrRNA sequences were identical to the sequences from the published genomes. The Neighbor-joining trees were generated in MEGA.^82^ Nucleotide diversity (Π) within housekeeping genes from *H. pylori* and *H. cetorum* were estimated in DnaSP,^83^ 95% confidence limits (Π_95_) were estimated using an online confidence limit calculator (https://www.statskingdom.com/confidence-interval-calculator.html). For the dating analyses, the four-gamete criterion implemented in DnaSP^83^ was used to identify stretches of DNA sequences that were likely to be the result of recombination. These recombinant nucleotides were removed from the dataset before the genetic distances between the groups were calculated in MEGA.^82^

### SDS-PAGE and Western blot

Bacteria were harvested and subsequently lysed by incubation in hot SDS buffer for 10 min. Protein extracts were separated on 8–12% SDS-PAGE gels and either Coomassie stained or blotted by Semi-dry blotting. Blotted PVDF membranes (Carl Roth, Karlsruhe, Germany) were analyzed using the following primary antibodies: rabbit α-Urease B (#21,987, BioGenes GmbH, Berlin, Germany),^38^ rabbit α-HtrA (#26823, BioGenes GmbH),^84^ rabbit α-VacA (#8036 BioGenes GmbH), rabbit α-GGT, rabbit α-FlaA, and rabbit α-NapA.^38^ Goat α-rabbit antibodies conjugated to horseradish peroxidase (Thermo Fisher Scientific) was used as secondary antibody.^52^ Chemiluminescence was detected using 1.41 mM luminol in 0.1 M Tris – HCL (pH 8.6) mixed with 0.61 mM p-coumaric acid in DMSO and 0.02% hydrogen peroxide. Bands were visualized using a ChemiDoc XRS + Gel Imaging System (Bio-Rad, Hercules, CA, USA).^52^

### Cultivation and infection of AGS cells and phase contrast microscopy

The epithelial cell line AGS (human gastric adenocarcinoma cells, ATCC #CRL-1739™) was cultured at 37°C in RPMI1640 medium containing 10% fetal bovine serum (Thermo Fisher Scientific) and antibiotics (1% penicillin/streptomycin and 0.2% normocin) in a humidified atmosphere supplemented with 5% CO_2_ as described.^52^ Before infection, cells were washed twice with phosphate-buffered saline (PBS, Sigma-Aldrich) and resuspended in fresh medium without antibiotics. The cells were then seeded into 6-well plates, grown to 70–80% confluency, and infected with *H. pylori* or *H. cetorum* at a multiplicity of infection (MOI) of 25. After 12 h, the samples were harvested and processed or analyzed by phase contrast microscopy with 10x objective to assess cell vacuolization. The frequency of cells containing vacuoles were expressed as mean values ± SEM after quantification of at least 100 cells per infection.

### NF-κB, IL-8 ELISA, activation of TLRs and PFGE

AGS cells cultured to confluency were transfected with 5 µg of the pNF-κB-SEAP reporter plasmid (http://www.addgene.org.) for 48 h using the TurboFect transfection reagent (Thermo Fisher Scientific), following the protocol of the supplier. NF-κB-dependent production of the secreted embryonic alkaline phosphatase (SEAP) was quantified before and after infection by incubation of 20 μL cell culture supernatant with 180 μL Quanti-Blue reagent (InvivoGen) for 30 min at 37°C. SEAP levels were estimated at OD_620_ in an Infinite F200 Pro microplate reader (Tecan, Grödig, Austria). IL-8 secretion from the infected cells was estimated by analyzing 20 µL of the supernatant in the IL-8 Human Uncoated ELISA Kit (Invitrogen, #88–8086). To monitor the activation of various TLRs (TLR1/2, TLR2, TLR4, TLR5, TLR9 and TLR10) by *H. pylori*, we used the corresponding HEK293 TLR/NF-κB/SEAP reporter cells (Invitrogen) in comparison to the HEK-Blue-Null1 cells as control. These 8 cell lines were infected for 24 h with either *H. pylori* or *H. cetorum* bacteria at a MOI of 25. The amount of NF-κB-dependent SEAP production was estimated as above by incubation of 180 µL Quanti-Blue reagent with 20 µL cell supernatant and subsequent measurement of the at OD_620_ in an Infinite F200 Pro microplate reader. To study the induction of chromosomal DSBs, AGS cells were infected for 8 h with *H. pylori* or *H. cetorum* strains at an MOI of 25. Cells were harvested, and total DNA was prepared and analyzed by PFGE as described.^58^

### Electron microscopy

For scanning electron microscopy (SEM), cultured *H. cetorum* bacteria were directly fixed in the growth medium by the addition of formaldehyde (final conc. 5%) and glutaraldehyde (final conc. 2%). Samples were centrifuged, washed twice with TE buffer (10 mM TRIS, 2 mM EDTA, pH 6.9), and dehydrated with stepwise increasing acetone concentrations (10, 30, 50, 70, 90%) on ice for 10 min each, followed by two incubation steps with 100% acetone for 10 min each. Samples were further processed via critical point drying with a CPD300 (Leica Microsystems), and sputter coated with gold/palladium using a SCD500 (Bal-Tec). The bacteria were examined in a field emission scanning electron microscope Merlin (Zeiss) using an acceleration voltage of 5 kV and both Inlens and an Everhart – Thornley SE detector. For negative staining, sample drops were pipetted on formvar coated grids and incubated for 45s. After soaking the liquid, the grids were washed twice in dH_2_0 followed by incubation on a droplet with uranyl-acetate for 30 sec and heat-drying on a bulb after removal of excessive liquid. The grids were examined in a transmission electron microscope Libra120 (Zeiss) at calibrated magnifications.

### Molecular modelling

The structure of hexameric *H. cetorum* VacA (MIT 99–5656) was predicted using AlphaFold-3,^85^ which resulted in a reliable model for residues I84-H896, with the exception of the amino acid residues D371-S408. The latter sequence stretch was also not resolved in the experimental structure of *H. pylori* VacA (PDB:6NYF)^67^ and was therefore excluded from further structural analysis. Structure comparison was done with PDBeFold,^86^ and RasMol^87^ was used for structure analysis and visualization.

### Statistics

All data were generated in at least three independent experiments and were evaluated by a two-tailed unpaired Student’s t-test and two-way ANOVA using the statistical analyses package implemented in Graph Pad Prism 8.0 (GraphPad Software, San Diego, CA, USA). Data are shown as mean values, and error bars indicate the standard error of the mean (SEM). The p-values of *p* ≤ 0.05 (*), *p* ≤ 0.01 (**) and *p* ≤ 0.001 (***) are considered to designate statistically significant results.

## Supplementary Material

Supplemental Figures.pdf

## Data Availability

All sequence sources are listed in the Materials and Methods section and are publicly available (https://www.ncbi.nlm.nih.gov/datasets/genome/?taxon=209), sequence alignments are provided in the supplementary material. The authors confirm that all data supporting the findings of this study are shown in the figures and are described in the text.
