# Supplementary material for: Molecular characterization of four Helicobacter cetorum strains from dolphins compared to human Helicobacter pylori
Source: Gut Microbes. 2025 Sep 25;17(1):2557982. doi: 10.1080/19490976.2025.2557982 (PMC12477871; doi:10.1080/19490976.2025.2557982)
Supplement: Supplemental Figures.pdf [file KGMI_A_2557982_SM1855.pdf]

Supplementary Figure S1

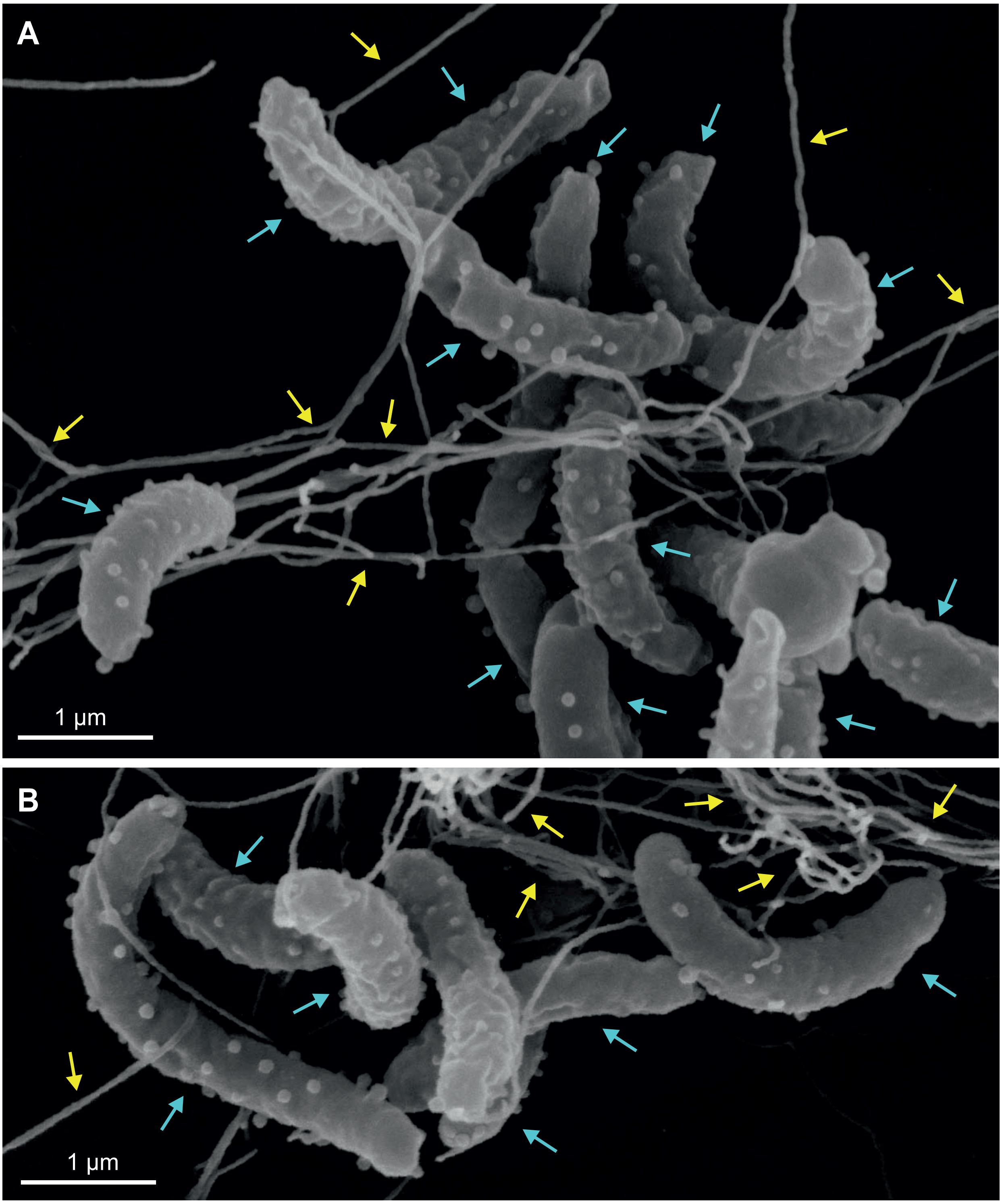

Supplementary Figure S2

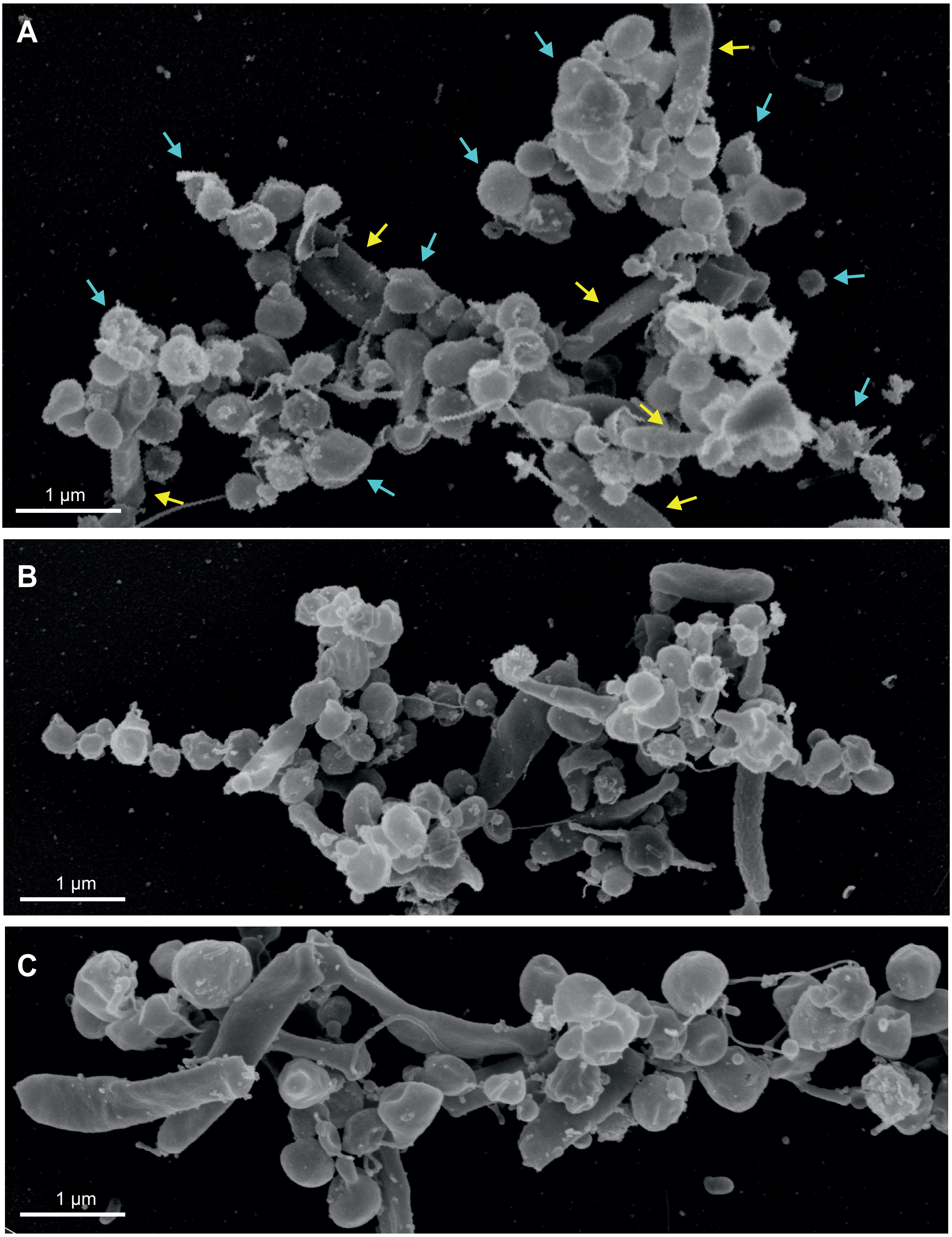

Supplementary Figure S3A

**Nickel co-factored urease**	
	1 … 100
**Hp_N6**	MKKISRKEYVSMYGPTTGDKVRLGDTDLIAEVEHDYTIYGEELKFGGGKTLREGMSQSNNPSKEELDLIITNALIVDYTGIYKADIGIKDGKIAGIGKGG
**Hp_G27**	MKKISRKEYVSMYGPTTGDKVRLGDTDLIAEVEHDYTIYGEELKFGGGKTLREGMSQSNNPSKEELDLIITNALIVDYTGIYKADIGIKDGKIAGIGKGG
**Hp_60190**	MKKISRKEYVSMYGPTTGDKVRLGDTDLIAEVEHDYTIYGEELKFGGGKTLREGMSQSNNPSKEELDLIITNALIVDYTGIYKADIGIKDGKIAGIGKGG
**Hp_P1**	MKKISRKEYVSMYGPTTGDKVRLGDTDLIAEVEHDYTIYGEELKFGGGKTLREGMSQSNNPSKEELDLIITNALIVDYTGIYKADIGIKDGKIAGIGKGG
**MIT99-5656**	MKKISRKEYASMYGPTTGDKVRLGDTELIAEVEHDYTIYGEELKFGGGKTLREGMSQSNNPSKEELDLIITNCLIVDYTGIYKADIGIKDGKIAGIGKGG
**MIT01-5903**	MKKISRKEYASMYGPTTGDKVRLGDTELIAEVEHDYTIYGEELKFGGGKTLREGMAQSNNPSKEELDLIITNALIVDYTGIYKADIGIKDGKIAGIGKGG
**MIT01-6096**	MKKISRKEYASMYGPTTGDKVRLGDTELIAEVEHDYTIYGEELKFGGGKTLREGMSQSNNPSKEELDLIITNCLIVDYTGIYKADIGIKDGKIAGIGKGG
**MIT01-6202**	MKKISRKEYASMYGPTTGDKVRLGDTELIAEVEHDFTIYGEELKFGGGKTLREGMSQSNNPSKEELDLIITNALIVDYTGIYKADIGIKDGKITGIGKGG
**Iron co-factored urease**	
**MIT99-5656**	MKKL---DYVNTYGPTKGDKVRLGDTELWAEVEHDYTIYGEELKFGAGKTIREGMGQSNSHDENTLDLVITNALIIDYTGIYKADIGIKNGKIHGIGKAG
**MIT01-6096**	MKKL---DYVNTYGPTKGDKVRLGDTELWAEVEHDYTIYGEELKFGAGKTIREGMGQSNSHDENTLDLVITNALIIDYTGIYKADIGIKNGKIHGIGKAG
**MIT01-6202**	MKKL---DYVNTYGPTKGDKVRLGDTELWAEVEHDYTIYGEELKFGAGKTIREGMGQSNSHDENTLDLVITNALIIDYTGIYKADIGIKNGKIHGIGKAG

**Nickel co-factored urease**	
	101 … 200
**Hp_N6**	NKDMQDGVKNNLSVGPATEALAGEGLIVTAGGIDTHIHFISPQQIPTAFASGVTTMIGGGTGPADGTNATTITPGRRNLKWMLRAAEEYSMNLGFLAKGN
**Hp_G27**	NKDMQDGVKNNLSVGPATEALAGEGLIVTAGGIDTHIHFISPQQIPTAFASGVTTMIGGGTGPADGTNATTITPGRRNLKWMLRAAEEYSMNLGFLAKGN
**Hp_60190**	NKDMQDGVKNNLSVGPATEALAGEGLIVTAGGIDTHIHFISPQQIPTAFASGVTTMIGGGTGPADGTNATTITPGRRNLKWMLRAAEEYSMNLGFLAKGN
**Hp_P1**	NKDMQDGVKNNLSVGPATEALAGEGLIVTAGGIDTHIHFISPQQIPTAFASGVTTMIGGGTGPADGTNATTITPGRRNLKWMLRAAEEYSMNLGFLAKGN
**MIT99-5656**	NKDMQDGVKNNLCVGPATEALAGEGLIVTAGGIDTHIHFISPQQIPTAFASGVTTMIGGGTGPADGTNATTITPGRRNLKWMLRAAEEYSMNLGFLGKGN
**MIT01-5903**	NKDMQDGVENNLCVGPATEALAAEGLIVTAGGIDTHIHFISPQQIPTAFASGVTTMIGGGTGPADGTNATTITPGRRNLKEMLRASEEYCMNLGFLGKGN
**MIT01-6096**	NKDMQDGVKNNLCVGPATEALAGEGLIVTAGGIDTHIHFISPQQIPTAFASGVTTMIGGGTGPADGTNATTITPGRRNLKWMLRAAEEYSMNLGFLGKGN
**MIT01-6202**	NKDMQDGVKNNLCVGPATEALAAEGLIVTAGGIDTHIHFISPQQIPTAFASGVTTMIGGGTGPADGTNATTITPGRRNLKWMLRAAEEYSMNLGFLGKGN
**Iron co-factored urease**	
**MIT99-5656**	NKDMQDGVSPNLVVGVGTEALAGEGMIITAGGIDSHTHFLSPQQFPTALANGVTTMFGGGTGPVDGTNATTITPGEWNIHRMLRAAEEYAMNVGFLGKGN
**MIT01-6096**	NKDMQDGVSPNLVVGVGTEALAGEGMIITAGGIDSHTHFLSPQQFPTALANGVTTMFGGGTGPVDGTNATTITPGVWNIHRMLRAAEEYAMNVGFLGKGN
**MIT01-6202**	NKDMQDGVSPNLVVGVGTEALAGEGMIITAGGIDSHTHFLSPQQFPTALANGVTTMFGGGTGPVDGTNATTITPGEWNIHRMLRAAEEYAMNVGLLGKGN

Nickel co-factored urease

	201 … 300
**Hp_N6**	ASNDASLADQIEAGAIGFKIHEDWGTTPSAINHALDVADKYDVQVAIHTDTLNEAGCVEDTMAAIAGRTMHTFHTEGAGGGHAPDIIKVAGEHNILPAST
**Hp_G27**	TSNDASLADQIEAGAIGFKIHEDWGTTPSAINHALDVADKYDVQVAIHTDTLNEAGCVEDTMAAIAGRTMHTFHTEGAGGGHAPDIIKVAGEHNILPAST
**Hp_60190**	ASNDASLADQIEAGAIGFKIHEDWGTTPSAINHALDVADKYDVQVAIHTDTLNEAGCVEDTMAAIAGRTMHTFHTEGAGGGHAPDIIKVAGEHNILPAST
**Hp_P1**	ASNDASLADQIEAGAIGFKIHEDWGTTPSAINHALDVADKYDVQVAIHTDTLNEAGCVEDTMAAIAGRTMHTFHTEGAGGGHAPDIIKVAGEHNILPAST
**MIT99-5656**	ASNDACLADQIEAGAIGFKIHEDWGTTPSAINHALDIADKYDVQVAIHTDTLNEAGCVEDTMAAIAGRTMHTFHTEGAGGGHAPDIIKVAGEHNILPAST
**MIT01-5903**	SSNDATLIDQLEAGACGFKIHEDWGTTPSAINHALDIADEYDVQVAIHTDTLNEAGCVEDTMEAIAGRTMHTFHTEGAGGGHAPDIIRVAGEHNILPAST
**MIT01-6096**	ASNDASLADQIEAGAIGFKIHEDWGTTPSAINHALDVADKYDVQVAIHTDTLNEAGCVEDTMAAIAGRTMHTFHTEGAGGGHAPDIIKVAGEHNILPAST
**MIT01-6202**	ASNDACLADQIEAGAIGFKIHEDWGTTPSAINHALDIADKYDVQVAIHTDTLNEAGCVEDTMAAIAGRTMHTFHTEGAGGGHAPDIIKVAGEHNILPAST

Iron co-factored urease

**MIT99-5656**	SSSKQQLVEQVHAGVIGFKLHEDWGTTPSAIDTCLSIADEYDVQVCIHTDTVNEAGYVNDTLNAMNGRAIHAYHIEGAGGGHSPDVITMAGEENILPSST
**MIT01-6096**	SSSKQQLVEQIEAGVIGFKLHEDWGTTPSAIDTCLSIADEYDVQVCIHTDTVNEAGYVNDTLNAMNGRAIHAYHIEGAGGGHSPDVITMAGEENILPSST
**MIT01-6202**	SSSKQQLVEQIEAGVVGFKLHEDWGTTPSAIDTCLGIADEYDVQVCIHTDTVNEAGYVNDTLNAMNGRAIHAYHIEGAGGGHSPDVITMAGEENILPSST

Nickel co-factored urease

	301 … 400
**Hp_N6**	NPTIPFTVNTEAEHMDMLMVCHHLDKSIKEDVQFADSRIRPQTIAAEDTLHDMGIFSITSSDSQAMGRVGEVITRTWQTADKNKKEFGRLKEE-KGDNDN
**Hp_G27**	NPTIPFTVNTEAEHMDMLMVCHHLDKSIKEDVQFADSRIRPQTIAAEDTLHDMGIFSITSSDSQAMGRVGEVITRTWQTADKNKKEFGRLKEE-KGDNDN
**Hp_60190**	NPTIPFTVNTEAEHMDMLMVCHHLDKSIKEDVQFADSRIRPQTIAAEDTLHDMGIFSITSSDSQAMGRVGEVITRTWQTADKNKKEFGRLKEE-KGDNDN
**Hp_P1**	NPTIPFTVNTEAEHMDMLMVCHHLDKSIKEDVQFADSRIRPQTIAAEDTLHDMGIFSITSSDSQAMGRVGEVITRTWQTADKNKKEFGRLKEE-KGDNDN
**MIT99-5656**	NPTIPFTVNTEAEHMDMLMVCHHLDKSIKEDVQFADSRIRPQTIAAEDALHDMGIFSITSSDSQAMGRVGEVITRTWQTADKNKKEFGRLKEE-KGDNDN
**MIT01-5903**	NPTIPFTKNTEAEHMDMLMVCHHLDKNIKEDVQFADSRIRPQTIAAEDVLHDMGIFSITSSDSQAMGRVGEVITRTWQTADKNKKEFGRLKEE-HGDNDN
**MIT01-6096**	NPTIPFTVNTEAEHMDMLMVCHHLDKSIKEDVQFADSRIRPQTIAAEDALHDMGIFSITSSDSQAMGRVGEVITRTWQTADKNKKEFGRLKEE-TGDNDN
**MIT01-6202**	NPTIPFTVNTEAEHMDMLMVCHHLDKSIKEDVQFADSRIRPQTIAAEDALHDMGIFSITSSDSQAMGRVGEVITRTWQTADKNKKEFGRLKEE-TGDNDN

Iron co-factored urease

**MIT99-5656**	TPTIPYTINTVAEHLDMLMTCHHLDKKIREDLQFSQSRIRPGSIAAEDVLHDNGMIAMTSSDSQAMGRAGEVVPRTWQTADKNKKEFGPLKEDAKNGNDN
**MIT01-6096**	TPTIPYTINTVAEHLDMLMTCHHLDKKIREDLQFSQSRIRPGSIAAEDVLHDNGMIAMTSSDSQAMGRAGEVVPRTWQTADKNKKEFGPLEEDAKNGNDN
**MIT01-6202**	TPTIPYTINTVAEHLDMLMTCHHLDKKIREDLQFSQSRIRPGSIAAEDVLHDNGMIAMTSSDSQAMGRAGEVVPRTWQTADKNKKEFGPLEEDAKNGNDN

	401 … 500
**Nickel co-factored urease**	
**Hp_N6**	FRIKRYLSKYTINPAIAHGISEYVGSVEVGKVADLVLWSPAFFGVKPNMIIKGGFIALSQMGDANASIPTPQPVYYREMFAHHGKAKYDANITFVSQAAY
**Hp_G27**	FRIKRYLSKYTINPAIAHGISEYVGSVEVGKVADLVLWSPAFFGVKPNMIIKGGFIALSQMGDANASIPTPQPVYYREMFAHHGKAKYDANITFVSQAAY
**Hp_60190**	FRIKRYLSKYTINPAIAHGISEYVGSVEVGKVADLVLWSPAFFGVKPNMIIKGGFIALSQMGDANASIPTPQPVYYREMFAHHGKAKYDANITFVSQAAY
**Hp_P1**	FRIKRYLSKYTINPAIAHGISEYVGSVEVGKVADLVLWSPAFFGVKPNMIIKGGFIALSQMGDANASIPTPQPVYYREMFAHHGKAKYDANITFVSQAAY
**MIT99-5656**	FRIKRYLSKYTINPAIAHGISEYVGSVEVGKVADLVLWSPAFFGVKPNMIIKGGFIALSQMGDANASIPTPQPVYYREMFAHHGKAKYDANITFVSQVAY
**MIT01-5903**	FRIKRYLSKYTINPAITHGISEYVGSVEVGKFADLVLWSPAFFGVKPNMIIKGGFIALSQMGDANASIPTPQPVYYREMFGHHGKAKFDGNITFVSQVAH
**MIT01-6096**	FRIKRYLSKYTINPAIAHGISEYVGSVEVGKVADLVLWSPAFFGVKPNMIIKGGFIALSQMGDANASIPTPQPVYYREMFAHHGKAKYDANITFVSQAAY
**MIT01-6202**	FRIKRYLSKYTINPAIAHGISEYVGSVEVGKVADLVLWSPAFFGVKPNMIIKGGFIALSQMGDANASIPTPQPVYYREMFAHHGKAKYDANITFVSQAAY
**Iron co-factored urease**	
**MIT99-5656**	FRIKRYISKYTINPAITHGVSEYIGSVETGKIADLVVWNPAFFGVKPKIIIKGGLVVFSEMGDSNASVPTPQPVYYREMFGHHGKAKFDTSITFVNKTAY
**MIT01-6096**	FRIKRYISKYTINPAITHGVSEYIGSVETGKIADLVVWNPAFFGVKPKIIIKGGLVVFSEMGDSNASVPTPQPVYYREMFGHHGKAKFDTSITFVNKLAY
**MIT01-6202**	FRIKRYISKYTINPAITHGVSEYIGSVETGKIADLVVWNPAFFGVKPKIIIKGGLVVFSEMGDSNASVPTPQPVYYREMFGHHGKAKFDTSITFVNKTAY

	501 … 570
**Nickel co-factored urease**	
**Hp_N6**	DKGIKEELGLERQVLPVKNCRNITKKDMQFNDTTAHIEVNPETYHVFVDGKEVTSKPANKVSLAQLFSIF
**Hp_G27**	DKGIKEELGLERQVLPVKNCRNITKKDMQFNDTTAHIEVNPETYHVFVDGKEVTSKPANKVSLAQLFSIF
**Hp_60190**	DKGIKEELGLERQVLPVKNCRNITKKDMQFNDTTAHIEVNPETYHVFVDGKEVTSKPANKVSLAQLFSIF
**Hp_P1**	DKGIKEELGLERQVLPVKNCRNITKKDMQFNDTTAHIEVNPETYHVFVDGKEVTSKPANKVSLAQLFSIF
**MIT99-5656**	ENGIKEELGLERQVLPVKNCRNITKKDMQFNDTTAHIEVNPETYHVHVDGKEVTSKAADKVSLAQLFSIF
**MIT01-5903**	DLGIKEELGLERHVLPVKNCRNITKKDLKFNDVTAHIEVNSETYKVKVDGKEVTSHAADEVSLAQLYSIF
**MIT01-6096**	ENGIKEELGLERQVLPVKNCRNITKKDMQFNDTTAHIEVNPETYHVHVDGKEVTSKAATEVSLAQLFSIF
**MIT01-6202**	ENGIKEELGLERQVLPVKNCRNITKKDMQFNDTTAHIEVNPETYHVHVDGKEVTSKAATEVSLAQLFNIF
**Iron co-factored urease**	
**MIT99-5656**	ENGIKEKLGLERKVLPIKNCRNVTKKDFKFNNTTGKLSVDPESFEVFLDGKLCSSKPASELPLAQRYTFF
**MIT01-6096**	ENGIKEKLGLERKVLPIKNCRNVTKKDFKFNNTTGKLSVDPESFEVFLDGKLCYSKPASELPLAQRYTFF
**MIT01-6202**	ENGIKEKLGLERKVLPIKNVRNITKKDFKFNNTTGKLSVDPESFEVFLDGKLCTSKPASELPLAQRYTFF

Supplementary Figure S3B

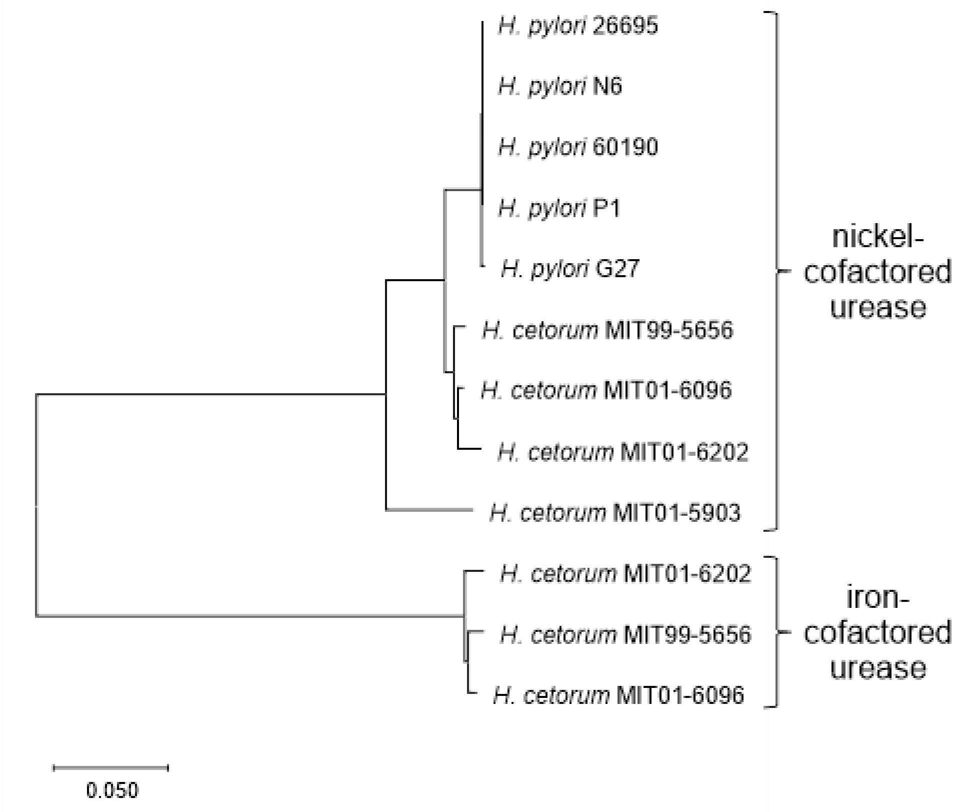

Supplementary Figure S4

Flagellin FlaA

```
            1                                                                                                  100
Hp_N6       MAFQVNTNINAMNAHVQSALTQNALKTSLERLSSGLRINKAADDASGMTVADSLRSQASSLGQAIANTNDGMGIIQVADKAMDEQLKILDTVKVKATQAA
Hp_G27      MAFQVNTNINAMNAHVQSALTQNALKTSLERLSSGLRINKAADDASGMTVADSLRSQASSLGQAIANTNDGMGIIQVADKAMDEQLKILDTVKVKATQAA
Hp_60190    MAFQVNTNINAMNAHVQSALTQNALKTSLERLSSGLRINKAADDASGMTVADSLRSQASSLGQAIANTNDGMGIIQVADKAMDEQLKILDTVKVKATQAA
Hp_P1       MAFQVNTNINAMNAHVQSALTQNALKTSLERLSSGLRINKAADDASGMTVADSLRSQASSLGQAIANTNDGMGIIQVADKAMDEQLKILDTVKVKATQAA
MIT99-5656  MAFQVNTNINALNAHVQSTLTQNALKTSLERLSSGLRINKAADDASGMTIADSLRSQASSLGQAIANTNDGMGIIQIADKAMDEQLKILDTVKVKATQAA
MIT01-5903  MAFQVNTNINALNAHVQSSLTQTALKNSLERLSSGLRINKAADDASGMTIADSLRSQASALGQAIANTNDGMGIIQIADKAMDEQLKILDTVKVKATQAA
MIT01-6096  MAFQVNTNINALNAHVQSSLTQTALKNSLERLSSGLRINKAADDASGMTIADSLRSQASALGQAIANTNDGMGIIQVADKAMDEQLKILDTVKVKATQAA
MIT01-6202  MAFQVNTNINALNAHVQSSLTQTALKNSLERLSSGLRINKAADDASGMTIADSLRSQASALGQAIANTNDGMGIIQVADKAMDEQLKILDTVKVKATQAA

          101                                                                                                  200
Hp_N6       QDGQTTESRKAIQSDIVRLIQGLDNIGNTTTYNGQALLSGQFTNKEFQVGAYSNQSIKASIGSTTSDKIGQVRIATGALITASGDISLTFKQVDGVNDVT
Hp_G27      QDGQTTESRKAIQSDIVRLIQGLDNIGNTTTYNGQALLSGQFTNKEFQVGAYSNQSIKASIGSTTSDKIGQVRIATGALITASGDISLTFKQVDGVNDVT
Hp_60190    QDGQTTESRKAIQSDIVRLIQGLDNIGNTTTYNGQALLSGQFTNKEFQVGAYSNQSIKASIGSTTSDKIGQVRIATGALITASGDISLTFKQVDGVNDVT
Hp_P1       QDGQTTESRKAIQSDIVRLIQGLDNIGNTTTYNGQALLSGQFTNKEFQVGAYSNQSIKASIGSTTSDKIGQVRIATGALITASGDISLTFKQVDGVNDVT
MIT99-5656  QDGQTTESRKAIQSDIVRLIQGLDNIGNTTTYNGQALLSGQFTNKEFQVGAYSNQSIKASIGSTTSDKIGQVRIATGSLITASGDISLTFRQVDGVNDVT
MIT01-5903  QDGQTTQSRKAIQADIVRLIQGLDNIGNTTTYNGQALLSGQFTNKEFQVGAYSNQSIKASIGSTTSDKIGQIRIATGALITASGDISLTFKQVDGVNDVK
MIT01-6096  QDGQTTQSRKAIQADIVRLIQGLDNIGNTTTYNGQALLSGQFTNKEFQVGAYSNQSIKASIGSTTSDKIGQVRIATGALITASGDVSLTFKQVDGVNDVK
MIT01-6202  QDGQTTQSRKAIQADIVRLIQGLDNIGNTTTYNGQALLSGQFTNKEFQVGAYSNQSIKASIGSTTSDKIGQVRIATGALITASGDVSLTFKQVDGVNDVK

          201                                                                                                  300
Hp_N6       LESVKVSSSAGTGIGVLAEVINKNSNRTGVKAYASVITTSDVAVQSGSLSNLTLNGIHLGNIADIKKNDSDGRLVAAINAVTSETGVEAYTDQKGRLNLR
Hp_G27      LESMKVSSSAGTGIGVLAEVINKNSNRTGVKAYASVITTSDVAVQSGSLSNLTLNGIHLGNIADIKKNDSDGRLVAAINAVTSETGVEAYTDQKGRLNLR
Hp_60190    LESVKVSSSAGTGIGVLAEVINKNSNRTGVKAYASVITTSDVAVQSGSLSNLTLNGIHLGNIADIKKNDSDGRLVAAINAVTSETGVEAYTDQKGRLNLR
Hp_P1       LESVKVSSSAGTGIGVLAEVINKNSNRTGVKAYASVITTSDVAVQSGSLSNLTLNGIHLGNIADIKKNDSDGRLVAAINAVTSETGVEAYTDQKGRLNLR
MIT99-5656  LESVKISSSAGTGIGVLAEVINKNSNRTGIRATANVITTSDTSVQSGNVSNLTLNGIHLGNIVAVKKNDTDGRLVAAINAVTSETGVEAYTDQDGRLNLR
MIT01-5903  LESVKISSSAGTGIGVLAEVINKNSNRTGVKAQANVITTSDDAIQSGNLSNLTLNGIHLGNIVDIKKNDSDGRLVAAINAVTSETGVEAYTDQNGRLNLR
MIT01-6096  LESVKISSSAGTGIGVLAEVINKNSDKTGIKAQANVITTSDDAIQSGSITNLTLNGIQLGNIVDIKKNDSDGRLVAAINAVTSDTGVEAYTDNKGRLNLR
MIT01-6202  LESVKISSSAGTGIGVLAEVINKNSDKTGIKAQANVITTSDDAIQSGSITNLTLNGIQLGNIVDIKKNDSDGRLVAAINAVTSDTGVEAYTDNKGRLNLR
```

	301 … 400
**Hp_N6**	SIDGRGIEIKTDSVSNG-PSALTMVNGGQDLTKGSTNYGRLSLTRLDAKSINVVSASDSQHLGFTAIGFGESQVAETTVNLRDVTGNFNANVKSASGANY
**Hp_G27**	SIDGRGIEIKTDSVSNG-PSALTMVNGGQDLTKGSTNYGRLSLTRLDAKSINVVSASDSQHLGFTAIGFGESQVAETTVNLRDVTGNFNANVKSASGANY
**Hp_60190**	SIDGRGIEIKTDSVSNG-PSALTMVNGGQDLTKGSTNYGRLSLTRLDAKSINVVSASDSQHLGFTAIGFGESQVAETTVNLRDVTGNFNANVKSASGANY
**Hp_P1**	SIDGRGIEIKTDSVSNG-PSALTMVNGGQDLTKGSTNYGRLSLTRLDAKSINVVSASDSQHLGFTAIGFGESQVAETTVNLRDVTGNFNANVKSASGANY
**MIT99-5656**	SIDGRGIDLKADKA-DGVPSALTTVNGGQDLTQGSTNYGRLSLSRLDAKSINVVSASDSQNMGFSAIGFGDSQVAETTVNLRDVTGNFNADVKSASGANY
**MIT01-5903**	SVDGRGIDIKADDNQDGSQVAIKNVNGGQDLTQGSTNYGRLSLSRLDARSISVVSASDSDKLGFTAIGFGQNQVAETTVNLRDVTGNFNANVKSASGANY
**MIT01-6096**	SVDGRGIDVKVDDNQDGSQIAIKNVNGGQELTQGSTNYGRLSLTRLDARDINVVSASDSSKIGFTAIGFGQNQVAETTVNLRDVTGNFNANVKSASGANY
**MIT01-6202**	SVDGRGIDVKVDDNQDGSQIAIKNVNGGQELTQGSTNYGRLSLTRLDARDINVVSASDSSKIGFTAIGFGQNQVAETTVNLRDVTGNFNANVKSASGANY

	401 … 500
**Hp_N6**	NAVIASGNQSLGSGVTTLRGAMVVIDIAESAMKMLDKVRSDLGSVQNQMISTVNNISITQVNVKAAESQIRDVDFAEESANFNKNNILAQSGSYAMSQAN
**Hp_G27**	NAVIASGNQSLGSGVTTLRGAMVVIDIAESAMKMLDKVRSDLGSVQNQMISTVNNISITQVNVKAAESQIRDVDFAEESANFNKNNILAQSGSYAMSQAN
**Hp_60190**	NAVIASGNQSLGSGVTTLRGAMVVIDIAESAMKMLDKVRSDLGSVQNQMISTVNNISITQVNVKAAESQIRDVDFAEESANFNKNNILAQSGSYAMSQAN
**Hp_P1**	NAVIASGNQSLGSGVTTLRGAMVVIDIAESAMKMLDKVRSDLGSVQNQMISTVNNISITQVNVKAAESQIRDVDFAEESANFNKNNILAQSGSYAMSQAN
**MIT99-5656**	NAVIASGNHSLGAGVTTLRGAMVVMDIAESAMKMLDKVRSDLGSVQNQMVSTVNNISITQVNVKAAESQIRDVDFAEESANFNKNNILAQSGSYAMSQAN
**MIT01-5903**	NAVIASGNHSLGAGVTTLRGAMVVMDVAESAMKMLDKVRSDLGSVQNQMISTVNNISITQVNVKAAESQIRDVDFADESANFNKNNILAQSGSYAMSQAN
**MIT01-6096**	NAVIASGNQSLGSGVTTLRGAMVVMDIAESATKMLDKVRSDLGSVQNQMISTVNNISITQVNVKAAESQIRDVDFADESANFNKNNILAQSGSYAMSQAN
**MIT01-6202**	NAVIASGNQSLGSGVTTLRGAMVVMDIAESATKMLDKVRSDLGSVQNQMISTVNNISITQVNVKAAESQIRDVDFADESANFNKNNILAQSGSYAMSQAN

	501 … 511
**Hp_N6**	TVQQNILRLLT
**Hp_G27**	TVQQNILRLLT
**Hp_60190**	TVQQNILRLLT
**Hp_P1**	TVQQNILRLLT
**MIT99-5656**	TVQQNILRLLT
**MIT01-5903**	TVQQNILRLLT
**MIT01-6096**	TVQQNILRLLT
**MIT01-6202**	TVQQNILRLLT

Supplementary Figure S5

Neutrophil-activating protein NapA

```
            1                                                                                                  100
Hp_N6       MKTFEILKHLQADAIVLFMKVHNFHWNVKGTDFFNVHKATEEIYEEFADMFDDLAERIAQLGHHPLVTLSEALKLTRVKEETKTSFHSKDIFKEILGDYK
Hp_G27      MKTFEILKHLQADAIVLFMKVHNFHWNVKGTDFFNVHKATEEIYEEFADMFDDLAERIAQLGHHPLVTLSEALKLTRVKEETKTSFHSKDIFKEILGDYK
Hp_60190    MKTFEILKHLQADAIVLFMKVHNFHWNVKGTDFFNVHKATEEIYEEFADMFDDLAERIAQLGHHPLVTLSEALKLTRVKEETKTSFHSKDIFKEILGDYK
Hp_P1       MKTFEILKHLQADAIVLFMKVHNFHWNVKGTDFFNVHKATEEIYEEFADMFDDLAERIAQLGHHPLVTLSEALKLTRVKEETKTSFHSKDIFKEILGDYK
MIT99-5656  MKTFEILRHLQADSIVLFMKVHNFHWNVKGHDFYHVHKATEEIYEEFAEMFDDVAERIVQLGHQPVVTLSEALKLACVKEETRTSFHSKEIFEEILKDYE
MIT01-5903  MKAVEILKHLQADSIVLFMKVHNFHWNVKGHDFYHVHKATEEIYEEFAEMFDDLAERIVQLGHHPVVTLSEAIKLAHVKEETKTSFHSKEIFEEILKDYE
MIT01-6096  MKTIEILKHLQADSIVLFMKVHNFHWNVKGHDFFHVHKATEEIYEEFAEMFDDVAERIVQLGHHPVVTLSEALKLAHVKEETKTSFHSKEIFEDILKDYE
MIT01-6202  MKTIEILKHLQADSIVLFMKVHNFHWNVKGHDFFHVHKATEEIYEEFAEMFDDVAERIVQLGHHPVVTLTEALKLAHVKEETKTSFHSKEIFEDILKDYE

           101                                        144
Hp_N6       HLEKEFKELSNTAEKEGDKVTVTYADDQLAKLQKSIWMLEAHLA
Hp_G27      HLEKEFKELSNTAEKEGDKVTVTYADDQLAKLQKSIWMLEAHLA
Hp_60190    HLEKEFKELSNTAEKEGDKVTVTYADDQLAKLQKSIWMLEAHLA
Hp_P1       HLEKEFKELSNTAEKEGDKVTVTYADDQLAKLQKSIWMLEAHLA
MIT99-5656  HLEKEFKKLSSVAEKDGDKVSVAYADEKLAKLEKSIWMLKAHLG
MIT01-5903  HLEKEFKKLSESAEKDGDKVTTAYADEQLAKLEKSIWMLKAHLG
MIT01-6096  HLLKEFKKLSEVAEKENDKVTIAYADEKLAKLEKSIWMLKAHLG
MIT01-6202  HLSKEFKKLSEVAEKENDKVTIAYADEKLAKLEKSIWMLKAHLG
```

Supplementary Figure S6

γ-glutamyltranspeptidase GGT

	1 – 100
**Hp_N6**	MRRSFLKTIGLGVIALSLGLLSPLSAASYPPIKNTKVGLALSSHPLATEIGQKVLEEGGNAIDAAVAMGFALAVVHPAAGNIGGGGFAVIHLANGENVAL
**Hp_G27**	MRRSFLKTIGLGVIVLSLGLLSPLSAASYPPIKNTKVGLALSSHPLATEIGQKILEDGGNAIDAAVAMGFALAVVHPAAGNIGGGGFAVIHLANGENVAL
**Hp_60190**	MRRSFLKTIGLGVIALSLGLLSPLSAASYPPIKNTKVGLALSSHPLATEIGQKVLEEGGNAIDAAVAMGFALAVVHPAAGNIGGGGFAVIHLANGENVAL
**Hp_P1**	MRRSFLKTIGLGVIALSLGLLSPLSAASYPPIKNTKVGLALSSHPLATEIGQKVLEEGGNAIDAAVAMGFALAVVHPAAGNIGGGGFAVIHLANGENVAL
**MIT99-5656**	MRRSFLKTIGLSVVALSLSLLSPLGAASYPPIKNTKVGLALSSHSLATEIGQKVLEDGGNAIDAAVAVGFALAVVHPAAGNIGGGGFAVIHLANGENVTL
**MIT01-5903**	MRRSFLKTIGLSAIALSLGLLNPLSSASYPPIKNAKVGLALSSHPLASEIGQKVLEEGGNAIDAAVAIGFALAVVHPAAGNIGGGGFAVIHLVNGENITL
**MIT01-6096**	MRRSFLKTISLGAIALSLGLLNPLSSASYPPIKNTKVGLALSSHPLASQVGQKVLEEGGNAIDAAVAVGFTLAVVHPAAGNIGGGGFAVIHLANGENITL
**MIT01-6202**	MRRSFLKTISLGAIALSLGLLNPLSSASYPPIKNTKVGLALSSHPLASQVGQKVLEEGGNAIDAAVAVGFALAVVHPAAGNIGGGGFAVIHLANGENITL

	101 – 200
**Hp_N6**	DFREKAPLKATKDMFLDKQGNVVPKLSEDGYLAAGVPGTVAGMEAMLKKYGTKKLSQLIDPAIKLAENGYAISQRQAETLKEARERFLKYSSSKKYFFKK
**Hp_G27**	DFREKAPLKATKNMFLDKQGNVVPKLSEDGYLAAGVPGTVAGMEAMLKKYGTKKLSQLIDPAIKLAENGYAISQRQAETLKEARERFLKYSSSKKYFFKK
**Hp_60190**	DFREKAPLKATKNMFLDKQGNVVPKLSEDGYLAAGVPGTVAGMEAMLKKYGTKKLSQLIDPAIKLAENGYAISQRQAETLKEARERFLKYSSSKKYFFKK
**Hp_P1**	DFREKAPLKATKNMFLDKQGNVVPKLSEDGYLAAGVPGTVAGMEAMLKKYGTKKLSQLIDPAIKLAENGYAISQRQAETLKEARERFLKYSSSKKYFFKK
**MIT99-5656**	DFREKAPLKATKDMFLDKQGNVVPKLSEDGYLAAGVPGTVAGMEAMLKKYGTKKLSQLIEPAIKLAERGYIISQRQAETLKEARERFLKYNSSKKYFLKK
**MIT01-5903**	DFREKAPLKATKDMYLDKQGNVIPKLSEDGYLAAGVPGTVAGMEAMLKKYGTKKLSRLIDPAIKLAEHGYVISQRQAETLKEAHERFNKYASSRKYFFKK
**MIT01-6096**	DFREKAPLKATRDMFLDKQGNVIPKLSEDGYLAAGVPGTVAGMEAMLKKYGTKKLSRLIDPAIKLAEHGYTISQRQAETLKEAHERFNKYASSKKYFFKK
**MIT01-6202**	DFREKAPLKATRDMFLDKQGNVIPKLSEDGYLAAGVPGTVAGMEAMLKKYGTKKLSRLIDPAIKLAEHGYTISQRQAETLKEAHERFNKYASSKKYFFKK

	201 – 300
**Hp_N6**	GHLDYQEGDLFVQKDLAKTLNQIKTLGAKGFYQGQVAELIEKDMKKNGGIITKEDLASYNVKWRKPVVGSYRGYKIISMSPPSSGGTHLIQILNVMENAD
**Hp_G27**	GHLDYQEGDLFVQKDLAKTLNQIKTLGAKGFYQGQVAELIEKDMKKNGGIITKEDLASYNVKWRKPVIGSYRGYKIISMSPPSSGGTHLIQILNVMENAD
**Hp_60190**	GHLDYQEGDLFVQKDLAKTLNQIKTLGAKGFYQGQVADLIEKDMKKNGGIITKEDLASYNVKWRKPVVGSYRGYKIISMSPPSSGGTHLIQILNVMENAD
**Hp_P1**	GHLDYQEGDLFVQKDLAKTLNQIKTLGAKGFYQGQVADLIEKDMKKNGGIITKEDLASYNVKWRKPVVGSYRGYKIISMSPPSSGGTHLIQILNVMENAD
**MIT99-5656**	GHLDYQEGDLFVQKDLAKTLTQIKLQGAKGFYHGHVAELIEKDMKKNGGIITKEDLAHYNVKWRKPVVGNYRGYKIISMSPPSSGGTHLIEILNVMENAD
**MIT01-5903**	GHHDYQEGDLFIQKDLAKTLTQIKLQGAKGFYHGHVAELIEKDMKKNGGIITQEDLANYKVKWRKPVVGTYRGYKIISMSPPSSGGTHLIEILNIMENAS
**MIT01-6096**	GHHDYQEGDLFIQRDLAKTLTQIKLQGAKGFYRGHTAELIEKDMKKNGGIITKEDLANYKVKWRKPVVGTYRGYKIISMSPPSSGGTHLIEILNVMENAD
**MIT01-6202**	GHHDYQEGDLFIQRDLAKTLTQIKLQGAKGFYRGHTAELIEKDMKKNGGIITKEDLANYKVKWRKPVVGTYRGYKIISMSPPSSGGTHLIEILNVMENAD

```
              301                                                                                                  400
Hp_N6         LSTLGYGASKNIHIAAEAMRQAYADRSVYMGDADFISVPVDKLINKAYAKKIFDTIQPDTVTPSSQIKPGMGQLHEGSNTTHYSVADRWGNAVSVTYTIN
Hp_G27        LSALGYGASKNIHIAAEAMRQAYADRSVYMGDADFVSVPVDKLINKAYAKKIFDTIQPDTVTPSSQIKPGMGQLHEGSNTTHYSVADRWGNAVSVTYTIN
Hp_60190      LSALGYGASKNIHIAAEAMRQAYADRSVYMGDADFVSVPVDKLINKAYAKKIFDTIQPDTVTPSSQIKPGMGQLHEGSNTTHYSVADRWGNAVSVTYTIN
Hp_P1         LSALGYGASKNIHIAAEAMRQAYADRSVYMGDADFVSVPVDKLINKAYAKKIFDTIQPDTVTPSSQIKPGMGQLHEGSNTTHYSVADRWGNAVSVTYTIN
MIT99-5656    LGALGYGASKNIHIAAEAMRQAYADRSVYMGDPDFVSVPVEKLTNKAYAKKIFDSIQPDTVTPSSQIKPGMGQLHEGNNTTHYSVADKWGNAVSITYTIN
MIT01-5903    LNEAGYGSSKNIHISAEAMRQAYADRSVYMGDPDFVQVPVEKLTSKLYAKKIFESIQPDTVTPSAKIKPGFNQLHEGNNTTHYSVADKWGNAVSVTYTIN
MIT01-6096    LSTQGFGSSKNIHITAEAMRQAYADRSVYMGDPDFVQVPVEKLTSKAYAKKIFESIQPDTVTPSAKIKPGFNQLHEGNNTTHYSVVDKWGNAVSVTYTIN
MIT01-6202    LSTQGFGSSKNIHIAAEAMRQAYADRSVYMGDPDFVQVPVEKLTSKAYAKKIFESIQLDTVTPSAKIKPGFNQLHEGNNTTHYSVVDKWGNAVSVTYTIN

              401                                                                                                  500
Hp_N6         ASYGSAASIDGAGFLLNNEMDDFSIKPGNPNLYGLVGGDANAIEANKRPLSSMSPTIVLKNNKVFLVVGSPGGSRIITTVLQVISNVIDYNMNISEAVSA
Hp_G27        ASYGSAASIDGAGFLLNNEMDDFSIKPGNPNLYGLVGGDANAIEANKRPLSSMSPTIVLKNNKVFLVVGSPGGSRIITTVLQVISNVIDYNMNVSEAVSA
Hp_60190      ASYGSAASIDGAGFLLNNEMDDFSIKPGNPNLYGLVGGDANAIEANKRPLSSMSPTIVLKNNKVFLVVGSPGGSRIITTVLQVISNVIDYNMNISEAVSA
Hp_P1         ASYGSAASIDGAGFLLNNEMDDFSIKPGNPNLYGLVGGDANAIEANKRPLSSMSPTIVLKNNKVFLVVGSPGGSRIITTVLQVISNVIDYNMNISEAVSA
MIT99-5656    ASYGSAASIDGAGFLLNNEMDDFSIKPGNPNLYGLVGGDANAIEANKRPLSSMSPTIVLKNNKVFMVVGSPGGARIITTVLQVISNVVDYDMNISEAVSA
MIT01-5903    ASYGSAASIDGAGFLLNNEMDDFSIKPGNPNLYGLVGGDANAIEASKRPLSSMSPTIVLKNNKVFMVVGSPGGARIITTVLQVISNVIDYNMNISEAVSA
MIT01-6096    ASYGSAAAIDGAGFLLNNEMDDFSIKPGNPNLYGLVGGDANAIEANKRPLSSMSPTIVLKNNKVFMVVGSPGGARIITTVLQVISNVIDYNMNISEAVSA
MIT01-6202    ASYGSAAAIDGAGFLLNNEMDDFSIKPGNPNLYGLVGGDANAIEANKRPLSSMSPTIVLKNNKVFMVVGSPGGARIITTVLQVISNVIDYNMNISEAVSA

              501                                                               567
Hp_N6         PRFHMQWLPDELRIEKFGMPADVKDNLTKMGYQIVTKPVMGDVNAIQVLPKTKGSVFYGSTDPRKEF
Hp_G27        PRFHMQWLPDELRIEKFGMPADVKDNLTKMGYQIVTKPVMGDVNAIQVLPKTKGSVFYGSTDPRKEF
Hp_60190      PRFHMQWLPDELRIEKFGMPADVKDNLTKMGYQIVTKPVMGDVNAIQVLPKTKGSVFYGSTDPRKEF
Hp_P1         PRFHMQWLPDELRIEKFGMPADVKDNLTKMGYQIVTKPVMGDVNAIQVLPKTKGSVFYGSTDPRKEF
MIT99-5656    PRFHMQWLPDELRIEKFGMPADVKESLTKMGYQIVTKPVMGDVNAIHIVPKTKGRIFYGATDPRKEF
MIT01-5903    PRFHMQWLPDELRIEKYGMPADVKDNLTKMGYQIVTKPVMGDVNAIHIKP--KSHVFYGATDPRKEF
MIT01-6096    PRFHMQWLPDELRIEKYGMPSDVKDNLTKMGYQIVTKPVMGDVNAILIKP--KGRVFYGATDPRKEF
MIT01-6202    PRFHMQWLPDELRIEKYGMPSDVKDNLTKMGYQIVTKPVMGDVNAILIKP--KGRVFYGATDPRKEF
```

Supplementary Figure S7

Serine protease HtrA

	1 … 100
**Hp_N6**	MMKKTLFISLALALSLNAGNIQIQSMPKVKERISVPSKDDTIYSYHDSIKDSIKAVVNISTEKKIKNNFIGGGVFNDPFFQQFFGDLGGMIPKERMERAL
**Hp_G27**	MMKKTLFISLALALSLNAGNIQIQNMPKVKERVSVPSKDDTIYSYHDSIKDSIKAVVNISTEKKIKNNFIGGGVFNDPFFQQFFGDLGGMIPKERMERAL
**Hp_60190**	MMKKTLFISLALALSLNAGNIQIQSMPKVKERVSVPSKDDTIYSYHDSIKDSIKAVVNISTEKKIKNNFIGGGVFNDPFFQQFFGDLGGMIPKERMERAL
**Hp_P1**	MMKKTLFISLALALSLNAGNIQIQSMPKVKERVSVPSKDDTIYSYHDSIKDSIKAVVNISTEKKIKNNFIGGGVFNDPFFQQFFGDLGGMIPKERMERAL
**MIT99-5656**	MMKKAFFISLTLALSLNAGSIQIQDMPKVKERVSVPSKDDTIYSYHDSIKNSIKAVVNISTEKKIKNSFMGGGMFSDPFFQQFFGDLGGMVPKERMERAL
**MIT01-5903**	MMKKAFFISLALALNLNAHNIEIQDMPKVKERVSVPSKDDTIYSYHDSIKDSIKAVVNISTEKKIKNNFMGGGMFSDPFFQQFFGDLGGAVPKDRIERAL
**MIT01-6096**	MMKKTLFISLVLALNLNANNIEIQDMPKIKERVSIPSKDDTIYSYHDSIKDSIKAVVNISTEKKIKNSFMGGGMFSDPFFQQFFGDLGGAVPKDRIERAL
**MIT01-6202**	MMKKTLFISLVLALNLNANNIEIQDMPKIKERVSIPSKDDTIYSYHDSIKDSIKAVVNISTEKKIKNSFMGGGMFSDPFFQQFFGDLGGAVPKDRIERAL

	101 … 200
**Hp_N6**	GSGVIISKDGYIVTNNHVIDGADKIKVTIPGSNKEYSATLVGTDSESDLAVIRITKDNLPTIKFSDSNDILVGDLVFAIGNPFGVGESVTQGIVSALNKS
**Hp_G27**	GSGVIISKDGYIVTNNHVIDGADKIKVTIPGSNKEYSATLVGTDSESDLAVIRITKDNLPTIKFSDSNDISVGDLVFAIGNPFGVGESVTQGIVSALNKS
**Hp_60190**	GSGVIISKDGYIVTNNHVIDGADKIKVTIPGSNKEYSATLVGTDSESDLAVIRITKDNLPTIKFSDSNDISVGDLVFAIGNPFGVGESVTQGIVSALNKS
**Hp_P1**	GSGVIISKDGYIVTNNHVIDGADKIKVTIPGSNKEYSATLVGTDSESDLAVIRITKDNLPTIKFSDSNDISVGDLVFAIGNPFGVGESVTQGIVSALNKS
**MIT99-5656**	GSGVIISKDGYIVTNNHVIDGADKIKVTIPGNNKEYSATLVGTDSESDLAVIRITKDNLPTIKFSDSNDILVGDLVFAIGNPFGVGESVTQGIISALNKS
**MIT01-5903**	GSGVIISKDGYIVTNNHVINEADKITVTIPGSTKEYSASLVGTDADSDLAVIRITKDNLPTIKFSDSNDILVGDLVFAIGNPFGVGETVTQGIVSALNKS
**MIT01-6096**	GSGVIISKDGYIVTNNHVINEADKITVTIPGSTKEYSASLVGTDADSDLAVIRINKDNLPTIKFSDSNDTLVGDLVFAIGNPFGVGETVTQGIVSALNKS
**MIT01-6202**	GSGVIISKDGYIVTNNHVINEADKITVTIPGSTKEYSASLVGTDADSDLAVIRINKDNLPTIKFSDSNDTLVGDLVFAIGNPFGVGETVTQGIVSALNKS

	201 … 300
**Hp_N6**	GIGINSYENFIQTDASINPGNSGGALIDSRGGLVGINTAIISKTGGNHGIGFAIPSNMVKDIVTQLIKTGKIERGYLGVGLQDLSGDLQNSYDNKEGAVV
**Hp_G27**	GIGINSYENFIQTDASINPGNSGGALIDSRGGLVGINTAIISKTGGNHGIGFAIPSNMVKDIVTQLIKTGKIERGYLGVGLQDLSGDLQNSYDNKEGAVV
**Hp_60190**	GIGINSYENFIQTDASINPGNSGGALIDSRGGLVGINTAIISKTGGNHGIGFAIPSNMVKDIVTQLIKTGKIERGYLGVGLQDLSGDLQNSYDNKEGAVV
**Hp_P1**	GIGINSYENFIQTDASINPGNSGGALIDSRGGLVGINTAIISKTGGNHGIGFAIPSNMVKDIVTQLIKTGKIERGYLGVGLQDLSGDLQNSYDNKEGAVV
**MIT99-5656**	GIGLNSYENFIQTDASINPGNSGGALIDSRGGLIGINTAILSKTGGNHGIGFAIPSNMVKDIVSQLIKTGKIERGYLGVGLQDVSSDLQNSYDNKEGAVV
**MIT01-5903**	GINLNNYENYIQTDASINPGNSGGALIDSRGGLIGINTAIISKTGGNHGIGFAIPSNMVKNIVSQLIKTGKIERGYLGVGLQDANNDLQSSYDGKEGAVV
**MIT01-6096**	GINLNNYENYIQTDASINPGNSGGALIDSRGGLIGINTAIISKTGGNHGIGFAIPSNMVKNIVSQLIKTGKIERGYLGVGLQDANNDLQSSYDGKEGAVV
**MIT01-6202**	GINLNNYENYIQTDASINPGNSGGALIDSRGGLIGINTAIISKTGGNHGIGFAIPSNMVKNIVSQLIKTGKIERGYLGVGLQDANNDLQSSYDGKEGAVV

```
           301                                                                                                  400
Hp_N6      ISVEKDSPAKKAGILVWDLITEVNGKKVKNTNELRNLIGSMLPNQRVTLKVIRDKKERTFTLTLAERKNPNKKETISAQNGAQGQLNGLQVEDLTQKTKR
Hp_G27     ISVEKDSPAKKVGILVWDLITEVNGKKVKNTNELRNLIGSMLPNQRVTLKVIRDKKERTFTLTLAERKNPNKKETISAQNGAQGQLNGLQVEDLTQKTKR
Hp_60190   ISVEKDSPAKKAGILVWDLITEVNGKKVKNTNELRNLIGSMLPNQRVTLKVIRDKKERTFTLTLAERKNPNKKETISAQNGAQGQLNGLQVEDLTQKTKR
Hp_P1      ISVEKDSPAKKAGILVWDLITEVNGKKVKNTNELRNLIGSMLPNQRVTLKVIRDKKERTFTLTLAERKNPNKKETISAQNGAQGQLNGLQVEDLTQKTKR
MIT99-5656 ISVEKDSPAKRAGILVWDLITEVNGKKIKNTNELRNLIGSMLPNQKVTLRVMRDKKERSFTLTLAERKNPNRKETSSTQGDAQGQLNGLKAEDLTPKTRK
MIT01-5903 ISVEKDSPAKRAGILVWDLITEVNGKKVKNSNELKNLIGSMLPNQRVTLKVIRDKKERTFTLTLAERKNPNKKETTSTQGNGQGQVSGLKVEDLTPKAKK
MIT01-6096 ISVEKDSPAKKAGLLVWDLITEVNGKKVKNSNELKNLIGSMLPNQKVTLKVIRDKKERFFTLILAERKNPNKKETTSTQGSTQGQVSGLKIEDLTPKTRK
MIT01-6202 ISVEKDSPAKKAGLLVWDLITEVNGKKVKNSNELKNLIGSMLPNQKVTLKVIRDKKERFFTLILAERKNPNKKETTSTQGSTQGQVSGLKIEDLTPKTRK

           401                                                                        476
Hp_N6      SMRLSDDVQGVLVSQVNENSPAEQAGFRQGNIITKIEEIEVKSVADFNHALEKYKGKPKRFLVLDLNQGYRIILVK
Hp_G27     SMRLSDDVQGVLVSQVNENSPAEQAGFRQGNIITKIEEIEVKSVADFNHALEKYKGKPKRFLVLDLNQGYRIILVK
Hp_60190   SMRLSDDVQGVLVSQVNENSPAEQAGFRQGNIITKIEEVEVKSVADFNHALEKYKGKPKRFLVLDLNQGYRIILVK
Hp_P1      SMRLSDDVQGVLVSQVNENSPAEQAGFRQGNIITKIEEVEVKSVADFNHALEKYKGKPKRFLVLDLNQGYRIILVK
MIT99-5656 SMRLGDDIQGVVIAQVNENSPAEQAGFRQGNIITRIEDVEIKSIADFNHALDKYKGKPKRFLILDLNQGYRLILVK
MIT01-5903 SMRLPDEIQGVIINQVKENSPAEQVGFRQGNIITRIEDVEIKNVANFNHALDKYKGKPKRFLIFDLNQGYRTILVK
MIT01-6096 SMRLPDEIQGVIISQVQENSPAEQAGFRQGNIITRIEDVEIKNVGNFNHALDKYKGKPKRFLIFDLNQGYRTIVVK
MIT01-6202 SMRLPDEIQGVIISQVQENSPAEQAGFRQGNIITRIEDVEIKNVGNFNHALDKYKGKPKRFLIFDLNQGYRTIVVK
```

Supplementary Figure S8

Vacuolating cytotoxin VacA

```
                1                     ◄───────────── S-region ─────────────►                       100
Hp_N6          MEIQQ---THRKINRPLVSLALVGALVSIT-----------------PQQSHAAF---------FTTVIIPAIVGGIATGTAVGTVSGLLSWGLKQAEEA
Hp_G27         MEIQQ---THRKMNRPLVSLVLAGALISAI-----------------PQESHAAF---------FTTVIIPAIVGGIATGTAVGTVSGLLSWGLKQAEEA
Hp_60190       MEIQQ---THRKINRPLVSLALVGALVSIT-----------------PQQSHAAF---------FTTVIIPAIVGGIATGTAVGTVSGLLGWGLKQAEEA
Hp_P1          MEKT-----HRKINRPLVSLALVGALVSIT-----------------PQQSHAAF---------FTTVIIPAIVGGIATGAAVGTVSGLLSWGLKQAEEA
MIT99-5656     MKSQK---KHRKMNRPIVSLALVGALVSAKLGANTPNNFQISTTTPPPYLQHAPI--HHNNYALLSGIIIPAIISGIASGIAAGTVGGIIGWVTKQAEQA
MIT01-5903     MSKST-QKIRHKMGRPVVSLALASILTSTSLSAQT-SHLEISPYAK-EHLQEAPIAPLDNNYGLLSD-ILKDVLGGIASGIASGAAAGIVSWITKQAEQA
MIT01-6096     MLKNQILKCPIKMSRPVVSLTLAGILTSTSLSANDTTNLEITPYAK-EHLQQAPF--INNNYGVWGD-ILKGIIGGIASGIGAGSAGGLIGWIIKQAENA
MIT01-6202     MLKNQILKCPIKMSRPVVSLALAGILTSTSLSANDITNLEITPYAK-EHLQQAPL--INNNYGVWGD-ILKGIIGGIASGIGAGSAGGLIGWIIKQAENA

              101                                                                                      200
Hp_N6          NKTPDKPDKVWRIQAGQGFNEFPNKEYDLYRSLLSSKIDGGWDWGNAARHYWVKGGQWNKLEVDMKDAVGTYTLSGLRNFTGGDLDVNMQKATLRLGQFN
Hp_G27         NKNPDKPDKVWRIQAGKGFNEFPNKEYDLYKSLLSSKIDGGWDWGNAARHYWVKGGQWNKLEVDMKDAVGTYKLSGLRNFTGGDLDVNMQKATLRLGQFN
Hp_60190       NKTPDKPDKVWRIQAGKGFNEFPNKEYDLYKSLLSSKIDGGWDWGNAATHYWIKGGQWNKLEVDMKDAVGTYKLSGLRNFTGGDLDVNMQKATLRLGQFN
Hp_P1          NKTPDKPEKVWRIQAGRGFDNFPHKQYDLYKSLLSSKIDGGWDWGNAARHYWVKDGQWNKLEVDMQNAVGTYNLSGLINFTGGDLDVNMQKATLRLGQFN
MIT99-5656     NKNPDKPHKIWRIKAGNGFDAFPDKQYDLYQSLLSTDIQSGWDWGNASNHFWVKDGQWNKLEVDMKNARGIYNLSGLINYTGGDLDVDMQRATLRLGQFN
MIT01-5903     SQAPNVPKKIWRVQAGLGFNLYPNKQYDLYQSLLSGNIQSGWDSGNASWHYWVRGGQWNKLEVNMKNARGIYNLSGLINYTGGDLDVDMQRATLRLGQFN
MIT01-6096     GEAPNVPEKIWRIKAGLGFNLYPNKQYDLYQSLLSADIQSGWAFGDAAWHYWVRGGQWNKLEVDMKTAVGTYNLSGLRNYTGGDLDVDMQKATLRLGQFN
MIT01-6202     GEAPNVPEKIWRIKAGLGFNLYPNKQYDLYQSLLSADIQSGWAFGDATWHYWVRGGQWNKLEVDMKSAVGTYNLSGLINYTGGDLDVDMQKATLRLGQFN

              201                                                                                      300
Hp_N6          GNSFTSYKDAADRTTRVDFNAKNISIDNFVEINNRVGSGAGRKASSTVLTLQASEGITSSKNAEISLYDGATLNLASN--------------SVKLMGNV
Hp_G27         GNSFTSYKDAADRTTRVNFNAKNISIDNFVEINNRVGSGAGRKASSTVLTLQASEGITSDKNAEISLYDGATLNLASS--------------SVKLMGNV
Hp_60190       GNSFTSYKDSADRTTRVDFNAKNILIDNFLEINNRVGSGAGRKASSTVLTLQASEGITSSKNAEISLYDGATLNLASN--------------SVKLNGNV
Hp_P1          GNSFTSFKDGANRTTRVDFNAKNILIDNFVEINNRVGSGAGRKASSTVLTLQASEKITSRENAEISLYDGATLNLVSSSN-----------HSVDLYGKV
MIT99-5656     GNSFTSFKDDNNRTTRVNFNAKNILIDNFIEINNRVGSGAGRKASSTVLTLQSSEGITSSENAEISLYDGATLNLISSWDRDLKANPWQAVRGVELKGNV
MIT01-5903     GNSFTSF---NERTTRVDFNANNILIDNFLEINNRVGDGLGRKPNATVLTLQSSEGITSSKNAEISLYNGATLNLISSWDKDFEANPWQIVRGVDLKGAV
MIT01-6096     GNSFTSF---NNRTTRVDFNAKNILIDNFLEINNRVGDGLGRKPNATVLTLQSQEGITSNNNAELYLYNGATLNLVSK--------------SVDFKGAV
MIT01-6202     GNSFTSF---NNRTTRVDFNAKNILIDNFLEINNRVGDGLGRKPNATVLTLQSQEGITSNNKAELYLYNGATLNLVSK--------------SVDFKGAV
```

```
           301                                                                                              400
Hp_N6       WMGRLQYVGAYLAPSYSTINTSKVTGEVNFNHLTVGDHNAAQAGIIASNKTHIGTLDLWQSAGLNIIAPPEGGYKDK-PKDKPSNTTQNN-----ANNNQ
Hp_G27      WMGRLQYVGAYLAPSYSTINTSKVTGEVNFNHLTVGDKNAAQAGIIASNKTHIGTLDLWQSAGLNIIAPPEGGYKDK-PNNTPSQSGTKNDKNESAKNDK
Hp_60190    WMGRLQYVGAYLAPSYSTINTSKVTGEVNFNHLTVGDHNAAQAGIIASNKTHIGTLDLWQSAGLNIIAPPEGGYKDK-PNNTPSQSG--------AKNDK
Hp_P1       WMGRLQYVGAYLAPSYSTINTSKVQGEMNFRHLAVGDRNAAQAGIIANKKLNIGTLDLWQSAGLNIIAPPEGGYESK-TKDNPQN---------------
MIT99-5656  WMGRLQYVGAYYAPSWSMIDTSQVKGDVIFHHLTVGDHNAAQAGIIASNKTKIGTLDLWQSAELHVIAPPEGGYKDKDKKHKGSSTQKNQ-----HNTEN
MIT01-5903  WMGRLQYPLAYSSPSWSLINTAEVYGRVNFSHLIVGDHNDAQAGIIANSNTNIGVLDLWQSAGLHIITPPQGGYKEEIAQNTQNSQEK-----------T
MIT01-6096  WMGRMQYPLAYSSPSWSMINTAEVNGKVNFNHLIIGDHNNAQAGIIANNDTNIGTLDLWQSAGLYVIAPPKGGYKKEFPQSTPNNQEK-------TAKKD
MIT01-6202  WMGRMQYPLAYLSPSWSMINTAEVNGKVNFNHLIIGDHNNAQAGIVANNDTNIGTLDLWQSAGLYVIAPPKGGYKKEFPQSTPNNQEK-------TAKKD

           401                                                                                              500
Hp_N6       QNSAQNNSNTQVINPPN---SAQKTETEPTQVIDGPFAGGKDTVVNINRINTNADGTIRAGGYKASLTTNAAHLHIGKGGINLSNQASGRTLLVENLTGN
Hp_G27      QESSQNNSNTQVINPPN---STQKTEIQPTQVIDGPFAGGKDTVVNINRINTNADGTIRVGGFKASLTTNAAHLHIGKGGVNLSNQASGRTLLVENLTGN
Hp_60190    QESSQNNSNTQVINPPN---STQKTEVQPTQVIDGPFAGGKDTVVNIDRINTKADGTIKVGGFKASLTTNAAHLNIGKGGVNLSNQASGRTLLVENLTGN
Hp_P1       -------------NPKN---DTQKTEIQPTQVIDGPFAGAKDTVVNIFRLNTNADGTIRVGGFKASLTTNAAHLHIGEGGINLSNQASGRSLLVENLTGN
MIT99-5656  TAKNDKDNNTQVINPSD----SQETEVQPVQVINGTFAGSKDSLVTIDHINVNSDTAVKVGGYKATLITNAENLNIGN--INLYNQASGRTLLVENQTGN
MIT01-5903  TKKNNSNNGVQVINPPN--SNSQKPQVEPVQVVNGPFPGAKNTVVNIYRLNVDTDASTKVGGYKAVLTTNAANLNIGQGGINLSNQASGRTLLVENQTGN
MIT01-6096  KSNNNNNNGVQVINAPNSSSNSQKVEVEPVQVINGPFPGAKNTVVNINRLNVDSDGTIRAGGFKAVLTTNAANLNIGD--VNIYNRASGRTLLVENETGN
MIT01-6202  KSNNNNNNGVQVINAPNSSSNSQKVEVEPVQVINGPFPGAKNTVVNINRLNVDSDGTIRAGGFKAVLTTNAANLNIGD--VNIYNRASGRTLLVENETGN

           501                                                              ◄──────────── m-region ────────────600
Hp_N6       ITVEGTLRVNNQVG--GAAVAGSSANFEFKAGEDTNNATATFNNDIHLGKAVNLRVDAHTAYFNGNVYLGKSTNLRVNGHSAHFKNIDASKSDNGLNTTT
Hp_G27      ITVDGPLRVNNQVG--GYALAGSSANFEFKAGVDTKNGTATFNNDISLGRFVNLKVDAHT---------------------ANFKGIDT--GNGGFNT--
Hp_60190    ITVDGPLRVNNQVG--GYALAGSSANFEFKAGVDTKNGTATFNNDISLGRFVNLKVDAHT---------------------ANFKGIDT--GNGGFNT--
Hp_P1       ITVEGALRVNNQVG--GSAIAGSSANFEFKAGADTNNGTATFNNDIHLGKAVNLRVDAHTAYFNGNIYLGKSTNLRVNGHSAHFKNIDATKSDNGLNTSA
MIT99-5656  ITVDGTLMVNNQIG--SYGFVGSGVNFIFKAGTDTHKGNATFNSNIYLGNSVNLKVDAKT---------------------ANFRDIDASKGNNSLNATT
MIT01-5903  ITVNGNLMVNNQVG--GFAFVGSRANFIFKAGTDTHNGIATFNSNIDLGNSVDLEVDAKT---------------------ANFKDINASNGNNSLNATT
MIT01-6096  ITINGPLMVNNQVGKYGFGFVGSSANFIFKAGTYSNHGIATFNSNIDLGSSVNLEVDAKV---------------------ANFKDINASNAHNGASANS
MIT01-6202  ITVNGPLMVNNQVGKYGFGFVGSSANFIFKAGTYSNHGIVTFNSNIDLGSSVNLEVDAKV---------------------ANFKDINASNAHNGASANS
```

```
          601 →                                                                                          700
Hp_N6        LDFSGVTDKVNINKLTTSATNVNIKNFDIKELVVTT-RVQSFGQYTIFGEDIGDKSRIGVVSLQTGYSPAYSGGVTFKSGKKLVIDEIYHAPWNYFDARN
Hp_G27       LDFSGVTDKVNINKLITASTNVAVKNFNINELIVKT-NGISVGEYTHFSEDIGSQSRINTVRLETGTRSIFSGGVKFKSGEKLVIDEFYYSPWNYFDARN
Hp_60190     LDFSGVTNKVNINKLITASTNVAVKNFNINELIVKT-NGVSVGEYTHFSEDIGSQSRINTVRLETGTRSIFSGGVKFKSGEKLVIDEFYYSPWNYFDARN
Hp_P1        LDLSGVTDKVNINKLTTSATNVNIKNFDIKELVVTT-RVQSFGQYTIFGENIGDKSRIGVVSLQTGYSPAYSGGVTFKSGKKLVIDEIYHAPWNYFDARN
MIT99-5656   LDFSGVTENIHINKLTTASTNVAAKNFNIKELDVTTSRGLSVGQYTDFTQDIGDKSHIETVRLETGYSPLYSGGIKFKSGKKLVIDEFYHAPWNYFDARN
MIT01-5903   LNFSGVSGMVNIDKLTTASTNVAVKNFHIKELYVTTSHGLSTGQYTYFSDDIGSNSVIDVVRLQKGYNPLYAGGIKFKKGEKLTIGSFYHAAWNYFDARN
MIT01-6096   LNFSNV-GMVNIDKLTTASTNVAVKNFHIKELYVTTSHGLSEGQYTYFGDDIGSKSVIDVVRLQRGYNPLYSGGVKFKKGEKLTIGSFYHAGWNYFDARK
MIT01-6202   LNFSNV-GMVNIDKLTTASTNVAVKNFHIKELYVTTSHGLSEGQYTYFGDDIGSKSVIDVVRLQRGYNPLYSGGVKFKKGEKLTIGSFYHAGWNYFDARK

          701                                                                                            800
Hp_N6        VTDVEVNKRILFGAPGNIAGKTGLMFNNLTLNSNASMDYGKDLDLTIQR---------------------------------------------------
Hp_G27       VKNVEITRKFASSTPENPWGTSKLMFNNLTLGQNAVMDYSQFSNLTIQGDFINNQGTINYLVRGGKVATLSVGNAAAMMFNNDIDSATGFYKPLIKINSA
Hp_60190     IKNVEITRKFASSTPENPWGTSKLMFNNLTLGQNAVMDYSQFSNLTIQGDFINNQGTINYLVRGGKVATLNVGNAAAMMFNNDIDSATGFYKPLIKINSA
Hp_P1        VTDVEINKRILFGAPGNIAGKTGLMFNNLTLNSNASMDYGKDLDLTIQGHFTNNQGTMNLFVQDGRVATLNAGHQASMIFNNLVDSATGFYKPLIKINNA
MIT99-5656   ITDVEITKKLLFAAPGSIVGMTGLMFNNLTLAKGANMDYGKDLDLTIQGNFTNNQGTMNILVQDGRFATLNVGQTATMKFNNMIDSATGFYKPLIKINDA
MIT01-5903   IREVVITKLLTFGAPGSIEGMTGLMFNNLTLAQGANMDYGKDLDLYIQGNFTNNQAVMNLNTQDGRVATLNVGNRATFDFNDNINSQTGFYNPLIKINYA
MIT01-6096   IKEVVITKLLTFGSPGSIEGMTGLMFNNLTLAKGANMDYGKDLDLYIQGNFTNNQAIMNLNTQDGRVATLNVGNRAIFDFNDNINSQTGFYNPLIKINDA
MIT01-6202   IKEVVITKLLTFGAPGSIEGMTGLMFNNLTLAKGANMDYGKDLDLYIQGNFTNNQAIMNLNTQDGRVATLNVGNRATFNFNDNINSQTGFYNPLIKINYA

          801                                                                                            900
Hp_N6        ------------------------------------------------------------------------GTAIGNQSMVNNPDSYKYLIGKAWKN
Hp_G27       QDLIKNTEHVLLKAKIIGYG-----NVSTGTNSISNVNLEEQFKERLALYNNN-NRMDTCVVR--NTD-DIKACGMAIGNQSMVNNPDNYKYLIGKAWKN
Hp_60190     QDLIKNTEHVLLKAKIIGYG-----NVSTGTNGISNVNLEEQFKERLALYNNN-NRMDTCVVR--NTD-DIKACGMAIGNQSMVNNPDNYKYLIGKAWKN
Hp_P1        QNLTKNKEHVLVRARNIDYNLVGVQGASYDNISASNTNLQEQFKERLALYNNN-NRMDICVVRKGNTD-DIKACGMAIGNQSMVNNPENYKYLEGKAWKN
MIT99-5656   QNLTKNVEHVLVKAQSIDYD-----NISTNT----DINQQEQFKERLALYNNH-NRMDICVVRKDSFESDVKACGMAIGNTDMVSHPDNYKYLEGRAWKN
MIT01-5903   DTLTKNVEHVLVSAQSIDYY-----NTSSNVNT--DIGQENAFKERIALYNSNHERMDICVVQ--DLN-DIRDCGMAIGNQNMVNDPSAYKYLEGRAWRN
MIT01-6096   NTLTKNVEHVLVSAQNIDYD-----NTSSNVNT--DINQESAFKERIALYNHN-ERMDICVVRQGDLN-DIRDCGIAIGNKKMVDDPSAYKYLEGRAWRN
MIT01-6202   NTLTKNVEHVLVSAQDIDYD-----NTSSNVNT--DINQESAFKERIALYNSNHERMDICVVQ--NLN-DIRDCGMAIGNKRMVSDPSAYKYLEGRAWRN
```

```
           901                                                                                                  1000
Hp_N6       TGINKTANNTTISVNLG-NNSAPTSSES--------NTTNLPTNTTNK--ARFASYALVKNAPFARYSATPNLVAINQHDFGTIESVFELANRSNDIDTL
Hp_G27      IGISKTANGSKISVYYL-GNSTPTENGG--------NTTNLPTNTTNN--ARSANYALVKNAPFA-HSATPNLVAINQHDFGTIESVFELANRSKDIDTL
Hp_60190    IGISKTANGSKISVYYL-GNSTPTENGG--------NTTNLPTNTTNN--ARFASYALIKNAPFA-HSATPNLVAINQHDFGTIESVFELANRSKDIDTL
Hp_P1       TGINKTADNTTIAVNLG-NNSAPTENGG--------NTTNLPTNTTNK--ARFASYALIKNAPFARYSATPNLVAINKHDFGTIESVFELANRSSDIDTL
MIT99-5656  TDIGKTATHKEIAVNVKDGHAAPKAHESTEEAKKDKNLISLPTNHNNVKLARFAHYALIRPNATTHSNTTPNLVAINKHNFGTIESVFELANRSDAINVI
MIT01-5903  TAIDKVATHKEIAISVQDSHIAPSATDS-------KDLINLPHSNENAQFARYANFSALN------SNATPNLVAINKNVFGTIESVFELANRYGAIESI
MIT01-6096  TAIDKVVTHKEIAVSVQDSHIAPNKNDS-------KDLINLPKNNENAKFAHYTNFATLN------SNATPHLVAINQNVFGTIESVFELANRFNAIKTI
MIT01-6202  TAIDKVVTHKEIAVSVQDSHIAPNAKDS-------KDFINLPHHNENEKFAHYTNFATLN------SNATPHLVAINQNVFGTIESVFELANRFNAIKTI

           1001                                                                                                 1100
Hp_N6       YANSGAQGRDLLQTLLIDSHDAGYARTMIDATSANEITKQLNTATTTLNN-IASLEHKTSGLQTLSLSNAMILNSRLVNLSRRHTNHIDSFAQRLQALKD
Hp_G27      YTHSGVQGRDLLQTLLIDSHDAGYARQMIDNTSTGEITKQLNAATDALNN-IASLEHKTSGLQTLSLSNAMILNSRLVNLSRKHTNHIDSFAQRLQALKG
Hp_60190    YANSGAQGRDLLQTLLIDSHDAGYARTMIDATSANEITKQLNTATTTLNN-IASLEHKTSSLQTLSLSNAMILNSRLVNLSRRHTNNIDSFAKRLQALKD
Hp_P1       YANSGAQGRDLLQTLLIDSHDAGYARTMIDATSANEITKQLNTATTTLNN-IASLEHKTSGLQTLSLSNAMILNSRLVNLSRRHTNNINSFAQRLQALKD
MIT99-5656  NATSGTQGRDLLQTLLIDSHNAGYARQMIDATSTGEITKQLNVATETLNN-IASLEHKTNSLQTLSLSNKMVVNTRLVNLSRKHTNNIDSFAKRLQALQD
MIT01-5903  KKDFGTQGRNLLQTMLINAHNAGYARQMIDANSTNTIIKDLGMATSVLNHQVNNLENKTSALQTLELSNAMTQNARLVNLSRRHTEYLSAFEQRLQALKN
MIT01-6096  KKDFGAQGRNLLQTMLIDAHNAGYARQMIDKTSTNTIIKDLNMATSVLNHQVANLENKTSALQTLELSNAMMQNARLVNLSRRHTKYLSAFEQRLQALKN
MIT01-6202  KKDFGAQGRNLLQTMLIDAHNAGYARQMIDKTSTNTIIKDLSMATSVLNHQVANLENKTSALQTLELSNAMTQNARLVNLSRRHTKYLSAFEQRLQALKN

           1101                                                                                                 1200
Hp_N6       QRFASLESAAEVLYQFAPKYEKPTNVWANAIGGASLNNGGNASLYGTSAGVDAYLNGQ-VEAIVGGFGSYGYSSFSNQANSLNSGANNTNFGVYSRIFAN
Hp_G27      QRFASLESAAEVLYQFAPKYEKPTNVWANAIGGASLNNGGNASLYGTSAGVDAYLNGE-VEAIVGGFGSYGYSSFSNRANSLNSGANNANFGVYSRIFAN
Hp_60190    QRFASLESAAEVLYQFAPKYEKPTNVWANAIGGASLNNGGNASLYGTSAGVDAYLNGQ-VEAIVGGFGSYGYSSFNNQANSLNSGANNTNFGVYSRIFAN
Hp_P1       QKFASLESAAEVLYQFAPKYEKPTNVWANAIGGTSLNNGGNASLYGTSAGVDAYLNGE-VEAIVGGFGSYGYSSFSNQANSLNSGANNTNFGVYSRIFAN
MIT99-5656  KRFASVGTMAEVLYQFAPKYEKPANVWANAIGGASLSNGGNTSLYGTSAGMDTYIEGENVEAIVGGFGSYGYSSFNNQASNLNSGANNTNFGLYSRVFAN
MIT01-5903  KRFSSVETMAEVLYKFAPKNEKHANIWANAIGGASLSNGGNTSLYGTSAGADTYIDGENLQAIVGGFGSYGYGS-QSGSSALNSWTNNTNFGLYSRVFSN
MIT01-6096  KRFSSVETMAEVLYKFAPKNESHANIWANAIGGASLSNGGNTSLYGTSAGADTYIEGENLQAIVGGFGSYGYGS-QSGSSALNSWTNNTNFGLYSRVFSN
MIT01-6202  KRFSSVETMAEVLYKFAPKNESHANIWANAIGGASLSNGGNTSLYGTSAGADTYIEGENLQAIVGGFGSYGYGS-QSGSSTLNSWTNNTNFGLYSRVFSN
```

```
            1201                                                                                            1300
Hp_N6       QHEFDFEAQGALGSDQSSLNFKSALLQDLNQSYNYLAYSAATRASYGYDFAFFRNALVLKPSVGVSYNHLGSTNFKSNSTNKVALSNGSSSQHLFNASAN
Hp_G27      QHEFDFEAQGALGSDQSSLNFKSALLQDLNQSYHYLAYSAATRASYGYDFAFFRNALVLKPSVGVSYNHLGSTNFKS-SSNQVALKNGSSSQHLFNANAN
Hp_60190    QHEFDFEAQGALGSDQSSLNFKSALLRDLNQSYNYLAYSAATRASYGYDFAFFRNALVLKPSVGVSYNHLGSTNFKSNSTNKVALSNGSSSQHLFNASAN
Hp_P1       QHEFDFEAQGALGSDQSSLNFKSALLQDLNQSYNYLAYSTATRASYGYDFAFFRNALVLKPSVGVSYNHLGSTNFKSNSNQKVALKNGASSQHLFNASAN
MIT99-5656  KHEFDFEAQGAVGSNNENLMFKGALLQGLNQGYSYLAYSAMANANYGYDFAFLNNALVFKPSVGVSYNHLGSTNMKS-NSNQVALSNGASSRHLLSANAN
MIT01-5903  KHEFDFNIQGAIGNNNESLMFKNLLLQGLNQGYNYMSYSTITQASYGYDFTFLNNALVLKPSVGVAYQNLGTTNIQS-HNAQLALSHGASNRNLFNINAN
MIT01-6096  KHEFDFNIQGAIGNNNENLMFKNALLQGLNQGYNYMAYSTIAQASYGYDFTFLNSALVLKPSVGVSYQNLGTTNIQS-HSTQLALSNGANNRNLFNINAN
MIT01-6202  KHEFDFNIQGAIGNNNESLMFKNSLLQGLNQGYNYMAYSTIAQASYGYDFTFLNSALVLKPSVGVSYQNLGTTNIQS-HSTQLALSNGANNRNLFNINAN

            1301                                                                                       1393
Hp_N6       VEARYYYGDTSYFYMNAGVLQEFANFGSSNAVSLNTFKVNAAHNPLNTHARVMMGGELKLAKEVFLNLGFVYLHNLISNIGHFASNLGMRYSF
Hp_G27      VEARYYYGDTSYFYMNAGVLQEFARFGSNNAASLNTFKVNTARNPLNTHARVMMGGELQLAKEVFLNLGVVYLHNLISNIGHFASNLGMRYSF
Hp_60190    VEARYYYGDTSYFYMNAGVLQEFANFGSSNAVSLNTFKVNATRNPLNTHARVMMGGELKLAKEVFLNLGVVYLHNLISNIGHFASNLGMRYSF
Hp_P1       VEARYYYGDTSYFYMNAGVLQEFAHVGSNNAASLNTFKVNATRNPLNTHARVMMGGELKLAKEVFLNLGFVYLHNLISNIGHFASNLGMRYSF
MIT99-5656  VEARYYYGDTSFFYMNAGILQELANFGFNNAMALNSFKVNATHNPLNTHARVMIGGELQLAKEVYLNLGVIYAHNLNTMIGNIASNLGMRYSF
MIT01-5903  LEARYYYGNTSYMYVNAGVLQEFANFGTNNAVSLNNFTIHASHSPLNTNARVMIGGELQLNKGVYLNLGLIYAHNFTLDMGSVASNLGMRYSF
MIT01-6096  LEARYYYGNTSYMYVNAGILQGFANFGTNNAVSLSNFAINASHSALNTNARVMIGGELQLNKGVYLNLGLIYAHNFTLDMGSIASNLGMRYSF
MIT01-6202  LEARYYYGNTSYMYVNAGILQGFANFGTNNAVSLSNFAINASHSALNTNARVMIGGELQLNKGVYLNLGLIYAHNFTLDRGSIASNLGMRYSF
```
